# Greenhouse agricultural plastic waste mapping database

**DOI:** 10.1016/j.dib.2020.106622

**Published:** 2020-12-07

**Authors:** Nicolas Afxentiou, Phoebe-Zoe Morsink Georgali, Angeliki Kylili, Paris A. Fokaides

**Affiliations:** a Frederick Research Center, Cyprus; b School of Engineering, Frederick University, 1036 Nicosia, Cyprus

**Keywords:** Greenhouses, Agricultural Plastic Waste (APW), Mapping, GIS software, Low- density polyethylene (LDPE)

## Abstract

Agricultural waste mapping is an indispensable tool for the development and adoption of sustainable waste management practises in the agricultural sector. Current practices of agricultural plastic waste (APW) management in countries with large agricultural areas and thus high generation volumes of APW include uncontrollable disposal in fields or near water sources, or uncontrolled burning of the waste. These practices lead to irreversible deterioration of the natural environment through land and soil contamination, contamination of freshwater resources, air pollution and also pose public health issues. Given these negative effects on the environment, spatial prediction of APW generation becomes significant in sustainable agriculture. This dataset consists of the coordinates of the agricultural plots identified in the Republic of Cyprus and the area in square meters covered by agricultural greenhouses. The dataset has been used to perform APW generation mapping and predict the national generation quantities of waste low- density polyethylene (LDPE). The collection of the data is included in sixteen tables separated per geographical area-cluster. The agricultural plastic waste (APW) generation mapping was conducted with the use of up-to-date statistics from Cyprus Agricultural Payments Organization (CAPO), geographic information system (GIS) and satellite imagery.

## Specifications Table

**Table:** 

Subject	Waste management
Specific subject area	Agricultural plastic waste (APW) mapping
Type of data	Tables; Figures
How data were acquired	Cyprus Agricultural Payments Organization (CAPO) for agricultural plots’ geographic locations ArcGIS tool for satellite imaging
Data format	Raw; Processed
Parameters for data collection	The geographic coordinates of the agricultural plots were obtained through the CAPO records. The land area covered by greenhouses was determined using ArcGIS. The LDPE specifications were obtained from personal communications with the Agricultural Research Institute (ARI).
Description of data collection	The CAPO records provided the geographic coordinates of all the plots, characterised as agricultural, nation- wide. Each of the plots was located with the use of ArcGIS, where satellite imaging helped in identifying the agricultural plots, which contained greenhouses and tunnels, as well as the plot area (in square meters) actually covered by greenhouses and tunnels. Based on information on the LDPE specifications used for greenhouses in Cyprus, obtained from questionnaires provided in relevant stakeholders, the potential generation quantities of APW were calculated.
Data source location	For obtaining the location allocation findings: Primary data sources: Cyprus Agricultural Payments Organization (CAPO) Available from: http://www.capo.gov.cy
Data accessibility	https://data.mendeley.com/datasets/3dpw885w4x/1

## Value of the Data

• The dataset shows the geographic locations of potential APW generation points and estimated quantities of APW generation on a national level
• The dataset regards the formation of clusters of agricultural plots containing greenhouses and tunnels and the geographic coordinates of the agricultural plot, the area of the plot and the area covered by greenhouses and/or tunnels within the plot and the potential quantity for APW generation for each plot.
• The dataset is useful for the development of supply chains and collection networks for APW, for drafting regional or national waste management plans for APW, as well as for decision-making regarding policies and incentives on waste management.

## 1 Data Description

**Fig. 1:**
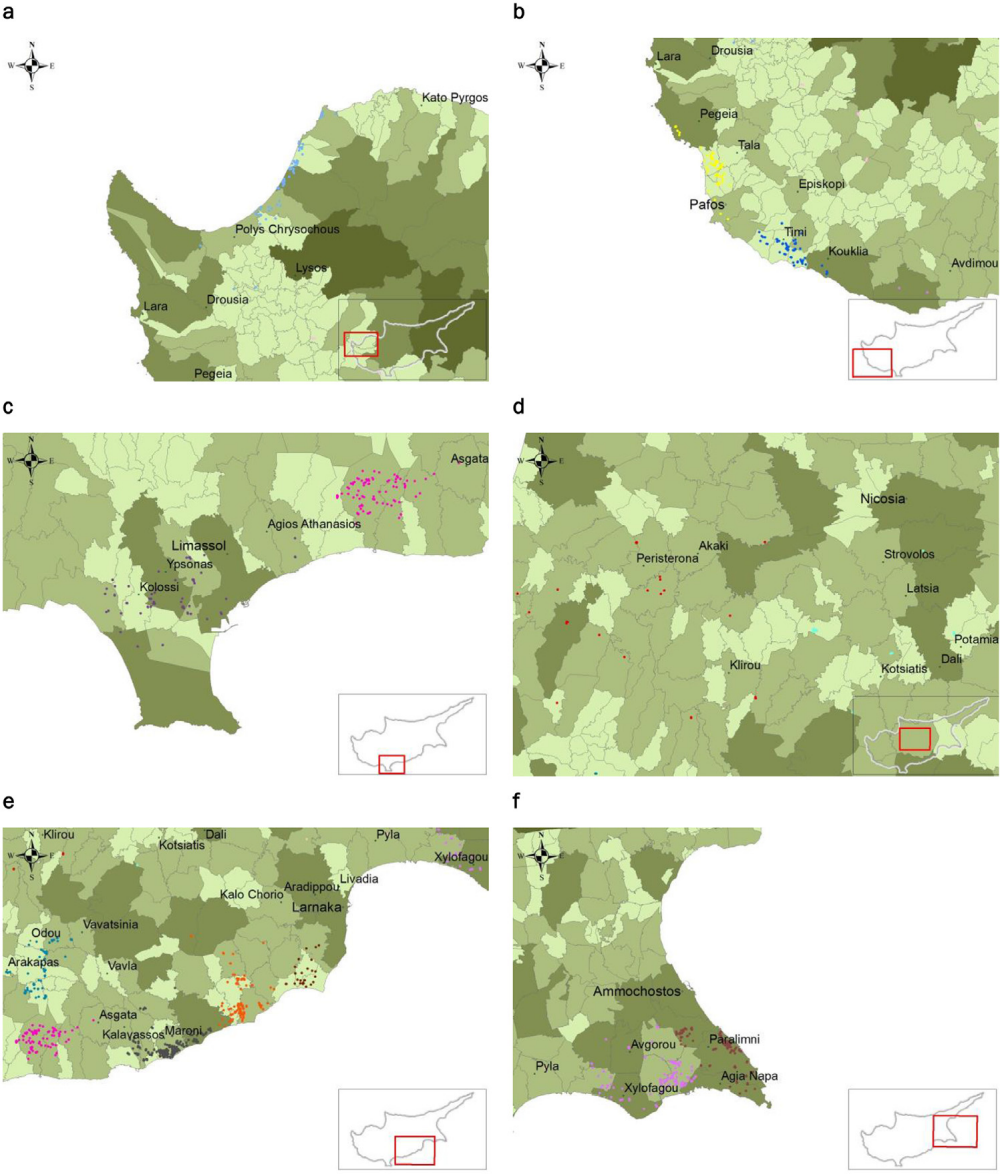
Geographic locations of greenhouses, distinguished into clusters: **a** – Cluster 1, **b** – Clusters 2, 3, 4, 5; **c** – Clusters 6, 8; **d** – Clusters 15, 16; **e** – Clusters 7, 9, 10, 11, 12; **f** – Clusters 13, 14.

The dataset is comprised of one (1) Table describing the clusters that have been created for the further analysis of this work (Table A0) and sixteen (16) Tables, providing data on the agricultural plots enclosing greenhouses and tunnels in Cyprus (Table A1–A16). The agricultural plots enclosing greenhouses and tunnels in Cyprus have been categorised in sixteen clusters, based on their geographic location and district they belong to. The geographic coordinates of the agricultural plots were obtained through personal communications with CAPO [1] and in the form of shape files that are provided in the repository. Each of the sixteen Tables (Table A1–A16) includes information and data of the agricultural plots included in the cluster. The Tables provide the plot identification (ID) numbers of the plots, as registered in the records of CAPO, their specific geographic coordinates (Longitude and Latitude), distinguish between the total plot area and the area covered by greenhouses or tunnels, as well as their estimated generated mass of APW (LDPE). The geographic locations of greenhouses in Cyprus, distinguished into the defined clusters, are shown in Fig. 1.

## 2 Experimental Design, Materials and Methods

The mapping of the greenhouse APW in Cyprus was conducted using primary data and information obtained from the records of CAPO on the latest statistics of recent years. In particular, the records of CAPO specify which agricultural plots enclose greenhouses and tunnels; the most recent records are for the year 2016. Accordingly, the number of plots enclosing greenhouses, their specific geographic locations and size are information that was deduced from the records. Given the exact coordinates of the agricultural plots, the plots have been located with the use of ArcGIS [2], a well- established and reliable GIS tool that enables gathering, managing, and analysing data, mapping and spatial reasoning. Based on the records, a total of 1160 agricultural plots are registered in Cyprus, of which 876 enclose greenhouses and tunnels. Each of these plots is assumed to be an APW generation source. Through the use of satellite imaging of the GIS tool, the actual area covered by greenhouses and/ or tunnels within the identified agricultural plots was determined. Based on these findings, the potential generation quantities of APW were calculated. Information taken into consideration for estimating the mass of agricultural plastic were obtained with the use of a questionnaire survey, conducted among relevant local stakeholders including the Agricultural Research Institute (ARI) [3], farm workers, agricultural land owners, farmers’ associations and greenhouse film manufacturers and importers. The findings of the questionnaire indicated that the four main types of greenhouses found in Cyprus include:

1. greenhouses of more than 3 m height
2. greenhouses of average height 2 – 3 m
3. tunnels of more than 3 m height
4. tunnels of less than 2 m height

The validation of the dataset and the characterization of the robustness of data has been implemented through the conduction of a comparative assessment with previous findings of research initiatives on quantities of agricultural plastics. Among the tasks under the research project ‘Design of a common agrochemical plastic packaging waste management scheme to protect natural resources in synergy with agricultural plastic waste valorisation – AgroChePack’, the area of greenhouses and plastic generated in the districts of Cyprus have been determined. The findings of the AgroChePack indicate to a total mass of 985,656 tonnes of generated plastics; a value which in comparison to the total mass of APW calculated in this work – 919,707 tonnes - is found among the acceptable limits of ± 10%.

The geographic locations of greenhouses in Cyprus distinguished into the defined clusters and were investigated in terms of this work are illustrated in Fig. 1.

## Ethics Statement

No ethical issues are associated with this work.

## Declaration of Competing Interest

The authors declare that they have no known competing financial interests or personal relationships which have, or could be perceived to have, influenced the work reported in this article.

## Appendix Supplementary materials

Image, application 1

## Footnotes

Supplementary material associated with this article can be found in the online version at doi:10.1016/j.dib.2020.106622.
