# Supplementary material for: Greenhouse agricultural plastic waste mapping database
Source: Data Brief. 2020 Dec 7;34:106622. doi: 10.1016/j.dib.2020.106622 (PMC7744705; doi:10.1016/j.dib.2020.106622)
Supplement: Supplementary file 1 [file mmc1.docx]

**Table A0**. Greenhouse Clusters in Cyprus

| **Cluster** | **Province** | **Location** |
| --- | --- | --- |
| 1 | Paphos | Northern area – Polis Chrysochous |
| 2 | Paphos | Western area – Pegeia |
| 3 | Paphos | Western area – Paphos urban area |
| 4 | Paphos | Southern area – Kouklia |
| 5 | Paphos | Mountainous area |
| 6 | Limassol | Southern area – Limassol urban area |
| 7 | Limassol | Eastern area – Parekklisia |
| 8 | Limassol | Mountainous area |
| 9 | Larnaca | Western area – Zygi |
| 10 | Larnaca | Southern area – Larnaca urban area |
| 11 | Larnaca | Mountainous area |
| 12 | Larnaca | Northern area – Athienou - Xilotimpou |
| 13 | Ammochostos | Eastern area |
| 14 | Ammochostos | Western area |
| 15 | Nicosia | Eastern area – Klirou |
| 16 | Nicosia | Western area – Dali |

**Table A1**. Records of Cyprus Agricultural Payments Organisation (CAPO) for the year 2016, provided in the form of shape files — Cluster 1

| **Plot ID** | **Longitude**  **[˚]** | **Latitude**  **[˚]** | **Plot Area**  **[m²]** | **Greenhouse Area**  **[m²]** | **LDPE mass**  **[kg]** |
| --- | --- | --- | --- | --- | --- |
| 788148 | 35,055045 | 32,488706 | 5892,58 | 2158,27 | 685,29 |
| 812118 | 35,107762 | 32,512350 | 1607,42 | 1042,89 | 331,14 |
| 974132 | 35,130590 | 32,522446 | 1495,05 | 1140,05 | 361,99 |
| 1072712 | 35,066657 | 32,470574 | 905,34 | 807,71 | 256,46 |
| 1141023 | 35,131272 | 32,522869 | 2353,92 | 929,77 | 295,22 |
| 1264485 | 35,091463 | 32,500062 | 5903,17 | 1010,21 | 320,76 |
| 774216 | 35,094443 | 32,500355 | 3714,14 | 2488,07 | 790,01 |
| 795342 | 35,064887 | 32,468918 | 6269,14 | 3661,98 | 1162,75 |
| 1069204 | 35,136620 | 32,518845 | 6103,48 | 4173,23 | 1325,09 |
| 1087855 | 35,114455 | 32,513996 | 1206,65 | 978,27 | 310,62 |
| 1096600 | 35,057749 | 32,469866 | 4020,69 | 520,70 | 165,33 |
| 1096628 | 35,086793 | 32,492418 | 9961,49 | 2212,66 | 702,56 |
| 1175941 | 35,062818 | 32,479826 | 21528,88 | 1142,91 | 362,90 |
| 1261860 | 35,024854 | 32,392334 | 13314,32 | 3755,79 | 1192,54 |
| 1267619 | 35,111540 | 32,514486 | 4584,41 | 833,38 | 264,61 |
| 905250 | 35,127824 | 32,519104 | 2644,20 | 1181,88 | 375,27 |
| 916901 | 35,130384 | 32,519698 | 2905,37 | 2049,65 | 650,81 |
| 957169 | 35,168764 | 32,549539 | 948,66 | 368,07 | 116,87 |
| 997529 | 35,107261 | 32,506980 | 3764,46 | 2864,56 | 909,55 |
| 1261858 | 35,024979 | 32,391604 | 14094,20 | 4114,12 | 1306,32 |
| 1265702 | 35,123478 | 32,523556 | 1837,20 | 1497,11 | 475,36 |
| 1298242 | 35,094219 | 32,498258 | 4758,18 | 1660,65 | 527,29 |
| 813564 | 35,113490 | 32,510021 | 2089,46 | 1704,45 | 541,20 |
| 814090 | 35,105107 | 32,512029 | 12828,70 | 799,97 | 254,01 |

**Table A1**. Records of Cyprus Agricultural Payments Organisation (CAPO) for the year 2016, provided in the form of shape files — Cluster 1 (cont’d)

| **Plot ID** | **Longitude**  **[˚]** | **Latitude**  **[˚]** | **Plot Area**  **[m²]** | **Greenhouse Area**  **[m²]** | **LDPE mass**  **[kg]** |
| --- | --- | --- | --- | --- | --- |
| 954255 | 35,130921 | 32,522511 | 4203,77 | 1410,89 | 447,99 |
| 970468 | 35,115693 | 32,517422 | 1922,43 | 1730,18 | 549,37 |
| 993386 | 35,109213 | 32,509526 | 5774,03 | 1130,75 | 359,04 |
| 998111 | 35,058877 | 32,468003 | 16352,44 | 952,38 | 302,40 |
| 1038271 | 35,099368 | 32,506477 | 2929,06 | 1430,21 | 454,12 |
| 1207082 | 35,166655 | 32,565833 | 2414,80 | 789,20 | 250,59 |
| 788165 | 35,103430 | 32,512772 | 12825,48 | 2575,97 | 817,92 |
| 798081 | 35,040961 | 32,437328 | 17168,70 | 663,79 | 210,77 |
| 819903 | 35,166369 | 32,548590 | 1829,57 | 737,70 | 234,23 |
| 832222 | 35,134031 | 32,524021 | 1716,07 | 877,48 | 278,62 |
| 835618 | 35,069781 | 32,490454 | 1037,69 | 933,93 | 296,54 |
| 885135 | 35,060553 | 32,463791 | 9866,18 | 4358,11 | 1383,79 |
| 930630 | 35,086976 | 32,493171 | 3689,86 | 1412,95 | 448,64 |
| 935302 | 35,128998 | 32,522034 | 6529,81 | 2740,92 | 870,30 |
| 939464 | 35,065258 | 32,483531 | 1204,61 | 1038,31 | 329,69 |
| 1077417 | 35,116681 | 32,516197 | 5504,63 | 656,51 | 208,46 |
| 1166538 | 35,058981 | 32,463487 | 5450,11 | 859,67 | 272,96 |
| 994676 | 35,164331 | 32,548851 | 2824,92 | 912,05 | 289,59 |
| 1109953 | 35,097293 | 32,500210 | 8546,00 | 1859,42 | 590,40 |
| 1143023 | 35,113633 | 32,516558 | 3893,47 | 1259,44 | 399,90 |
| 1156022 | 35,058562 | 32,470127 | 13362,85 | 541,33 | 171,88 |
| 1210389 | 35,163128 | 32,546585 | 813,28 | 624,91 | 198,42 |
| 1237844 | 35,108170 | 32,506919 | 2094,93 | 749,14 | 237,87 |
| 785508 | 35,089781 | 32,498031 | 5998,43 | 4692,59 | 1489,99 |
| 811779 | 35,093474 | 32,497607 | 10236,54 | 1257,54 | 399,29 |
| 814381 | 35,066895 | 32,479733 | 1571,10 | 1130,83 | 359,06 |
| 889870 | 35,164402 | 32,549471 | 3483,76 | 1798,09 | 570,93 |
| 1091263 | 35,068426 | 32,495111 | 2796,17 | 2516,55 | 799,06 |
| 1148541 | 35,092414 | 32,499780 | 6685,04 | 2110,34 | 670,07 |
| 813563 | 35,108851 | 32,507377 | 3344,58 | 614,50 | 195,12 |
| 880659 | 35,053984 | 32,476286 | 5798,91 | 1133,05 | 359,77 |
| 1084953 | 35,087404 | 32,492155 | 4223,43 | 3765,34 | 1195,57 |

**Table A2**. Records of Cyprus Agricultural Payments Organisation (CAPO) for the year 2016, provided in the form of shape files — Cluster 2

| **Plot ID** | **Longitude**  **[˚]** | **Latitude**  **[˚]** | **Plot Area**  **[m²]** | **Greenhouse Area**  **[m²]** | **LDPE mass**  **[kg]** |
| --- | --- | --- | --- | --- | --- |
| 818537 | 34,764565 | 32,416017 | 6564,29 | 3998,88 | 1269,72 |
| 936644 | 34,824657 | 32,418118 | 4887,74 | 3274,45 | 1039,70 |
| 1280557 | 34,812315 | 32,411894 | 7902,70 | 3543,61 | 1125,17 |
| 791144 | 34,838757 | 32,396082 | 5272,88 | 2667,85 | 847,10 |

**Table A2**. Records of Cyprus Agricultural Payments Organisation (CAPO) for the year 2016, provided in the form of shape files — Cluster 2 (cont’d)

| **Plot ID** | **Longitude**  **[˚]** | **Latitude**  **[˚]** | **Plot Area**  **[m²]** | **Greenhouse Area**  **[m²]** | **LDPE mass**  **[kg]** |
| --- | --- | --- | --- | --- | --- |
| 791156 | 34,827884 | 32,420339 | 13898,74 | 5431,24 | 1724,53 |
| 1074503 | 34,841130 | 32,403285 | 9053,58 | 7437,14 | 2361,44 |
| 1186186 | 34,829582 | 32,411755 | 1925,28 | 870,21 | 276,31 |
| 1250122 | 34,758549 | 32,428199 | 13732,54 | 1601,92 | 508,64 |
| 1294537 | 34,827719 | 32,401042 | 6125,00 | 1723,29 | 547,18 |
| 1308165 | 34,847458 | 32,386457 | 8571,82 | 5546,98 | 1761,28 |
| 788101 | 34,803368 | 32,415056 | 1614,00 | 682,83 | 216,81 |
| 790695 | 34,832263 | 32,403334 | 3562,09 | 2892,35 | 918,38 |
| 790846 | 34,831296 | 32,391339 | 1117,07 | 909,78 | 288,87 |
| 791157 | 34,829541 | 32,410048 | 3377,80 | 1503,55 | 477,41 |
| 930689 | 34,834217 | 32,404312 | 2036,16 | 1513,11 | 480,44 |
| 937163 | 34,823175 | 32,417882 | 8166,32 | 1488,59 | 472,66 |
| 997041 | 34,802780 | 32,413039 | 9609,42 | 4029,24 | 1279,36 |
| 1087086 | 34,831382 | 32,408330 | 7976,52 | 2840,07 | 901,78 |
| 1133394 | 34,873409 | 32,347409 | 2432,51 | 598,79 | 190,13 |
| 1155947 | 34,825201 | 32,407933 | 5358,28 | 3948,26 | 1253,65 |
| 789821 | 34,805239 | 32,419736 | 11032,11 | 3596,78 | 1142,05 |
| 867269 | 34,829362 | 32,409701 | 3183,77 | 2382,15 | 756,38 |
| 931503 | 34,873529 | 32,347862 | 1367,55 | 1179,39 | 374,48 |
| 1010543 | 34,873821 | 32,348423 | 1323,66 | 993,81 | 315,55 |
| 1256892 | 34,808895 | 32,420188 | 7969,37 | 998,99 | 317,20 |
| 787504 | 34,822414 | 32,414695 | 3202,22 | 993,95 | 315,60 |
| 787532 | 34,828934 | 32,411128 | 2614,88 | 1247,47 | 396,10 |
| 788092 | 34,830507 | 32,413316 | 2373,49 | 2136,14 | 678,27 |
| 839497 | 34,866119 | 32,353857 | 10026,55 | 527,72 | 167,56 |
| 790526 | 34,801626 | 32,413814 | 3919,07 | 1320,40 | 419,25 |
| 791588 | 34,838141 | 32,396751 | 6846,99 | 1015,45 | 322,43 |
| 1141941 | 34,836199 | 32,402642 | 7946,71 | 4418,36 | 1402,92 |
| 1256200 | 34,803046 | 32,410953 | 12335,55 | 1485,39 | 471,64 |
| 787565 | 34,821323 | 32,411940 | 3965,69 | 1023,44 | 324,96 |
| 788196 | 34,826460 | 32,404667 | 3609,36 | 709,33 | 225,23 |
| 791891 | 34,826054 | 32,408273 | 3539,83 | 2232,22 | 708,77 |
| 925313 | 34,824115 | 32,394812 | 1533,36 | 1373,10 | 435,99 |
| 1099221 | 34,834861 | 32,402948 | 3383,74 | 1843,75 | 585,43 |
| 1165828 | 34,811672 | 32,417226 | 4795,84 | 4052,29 | 1286,68 |
| 791004 | 34,813379 | 32,410059 | 2790,43 | 534,73 | 169,79 |
| 791713 | 34,803602 | 32,412613 | 3789,70 | 1503,10 | 477,26 |
| 867443 | 34,814750 | 32,412353 | 7284,66 | 1027,72 | 326,32 |
| 885779 | 34,863357 | 32,353616 | 4521,72 | 404,46 | 128,42 |
| 1087202 | 34,840541 | 32,405787 | 5423,57 | 2509,94 | 796,96 |
| 1099155 | 34,837836 | 32,402056 | 5740,56 | 3429,24 | 1088,85 |

**Table A3**. Records of Cyprus Agricultural Payments Organisation (CAPO) for the year 2016, provided in the form of shape files — Cluster 3

| **Plot ID** | **Longitude**  **[˚]** | **Latitude**  **[˚]** | **Plot Area**  **[m²]** | **Greenhouse Area**  **[m²]** | **LDPE mass**  **[kg]** |
| --- | --- | --- | --- | --- | --- |
| 803786 | 34,706600 | 32,539712 | 4836,76 | 3147,23 | 999,31 |
| 818537 | 34,764565 | 32,416017 | 6564,29 | 4550,69 | 1444,94 |
| 856819 | 34,705754 | 32,540160 | 10508,04 | 5349,65 | 1698,62 |
| 894952 | 34,691675 | 32,578403 | 9932,41 | 7172,87 | 2277,53 |
| 939934 | 34,706441 | 32,539014 | 3066,86 | 2760,17 | 876,41 |
| 1045026 | 34,728634 | 32,528952 | 4338,29 | 2739,75 | 869,93 |
| 1056024 | 34,721215 | 32,529926 | 1057,77 | 952,00 | 302,28 |
| 1105581 | 34,722430 | 32,517323 | 2223,74 | 881,35 | 279,85 |
| 1145050 | 34,728750 | 32,511024 | 2512,60 | 2261,34 | 718,02 |
| 1267377 | 34,728558 | 32,515839 | 3992,33 | 2787,38 | 885,05 |
| 814720 | 34,733853 | 32,501662 | 1979,82 | 723,15 | 229,62 |
| 819356 | 34,728053 | 32,486704 | 14363,17 | 3023,83 | 960,13 |
| 861371 | 34,703286 | 32,541018 | 7692,15 | 7279,62 | 2311,42 |
| 910373 | 34,721864 | 32,517163 | 2269,63 | 1996,43 | 633,91 |
| 931952 | 34,721323 | 32,529652 | 3102,17 | 2800,87 | 889,33 |
| 988606 | 34,697722 | 32,572275 | 403663,74 | 10221,48 | 3245,53 |
| 1006461 | 34,721057 | 32,518109 | 1499,05 | 975,91 | 309,87 |
| 1094820 | 34,721969 | 32,516144 | 5837,91 | 1051,23 | 333,79 |
| 1117624 | 34,690799 | 32,578006 | 8631,30 | 7084,11 | 2249,35 |
| 1250122 | 34,758549 | 32,428199 | 13732,54 | 22473,79 | 7135,88 |
| 856713 | 34,722224 | 32,537598 | 3792,04 | 3060,33 | 971,72 |
| 891946 | 34,710479 | 32,544362 | 3531,22 | 1192,65 | 378,69 |
| 911121 | 34,714802 | 32,514852 | 8106,54 | 4508,71 | 1431,61 |
| 938683 | 34,721076 | 32,529259 | 3729,26 | 2915,42 | 925,70 |
| 985097 | 34,736789 | 32,480774 | 3240,93 | 2697,20 | 856,41 |
| 1117947 | 34,724601 | 32,519961 | 3689,80 | 380,54 | 120,83 |
| 1121380 | 34,719953 | 32,526451 | 5094,61 | 4525,51 | 1436,94 |
| 1130822 | 34,753526 | 32,509874 | 2069,96 | 899,16 | 285,50 |
| 918162 | 34,728358 | 32,530657 | 5152,31 | 1666,88 | 529,27 |
| 930272 | 34,719834 | 32,527068 | 1915,05 | 1033,82 | 328,26 |
| 930688 | 34,711291 | 32,523761 | 2438,50 | 1216,52 | 386,27 |
| 954051 | 34,730967 | 32,502477 | 2691,44 | 1663,69 | 528,26 |
| 970011 | 34,703040 | 32,545765 | 19389,71 | 9850,58 | 3127,76 |
| 994421 | 34,753649 | 32,509937 | 1309,10 | 602,87 | 191,42 |
| 1005832 | 34,710877 | 32,524006 | 2720,80 | 2286,44 | 725,99 |
| 1051883 | 34,737501 | 32,478844 | 6476,50 | 2065,01 | 655,68 |
| 782559 | 34,728891 | 32,530478 | 3245,15 | 964,31 | 306,19 |
| 930892 | 34,720394 | 32,518566 | 6297,48 | 3806,10 | 1208,51 |
| 932261 | 34,719751 | 32,527434 | 4560,63 | 3560,26 | 1130,45 |
| 927611 | 34,733403 | 32,502467 | 683,46 | 260,56 | 82,73 |
| 1011216 | 34,720881 | 32,529856 | 1396,82 | 1081,65 | 343,45 |

**Table A3**. Records of Cyprus Agricultural Payments Organisation (CAPO) for the year 2016, provided in the form of shape files — Cluster 3 (cont’d)

| **Plot ID** | **Longitude**  **[˚]** | **Latitude**  **[˚]** | **Plot Area**  **[m²]** | **Greenhouse Area**  **[m²]** | **LDPE mass**  **[kg]** |
| --- | --- | --- | --- | --- | --- |
| 1036726 | 34,720864 | 32,528860 | 6318,48 | 4835,03 | 1535,22 |
| 1048840 | 34,731778 | 32,505477 | 3156,55 | 2645,93 | 840,14 |
| 1116457 | 34,732085 | 32,527474 | 1886,42 | 1689,70 | 536,51 |
| 1139355 | 34,727931 | 32,510786 | 2351,17 | 2116,06 | 671,89 |
| 1185092 | 34,720575 | 32,519393 | 2624,24 | 1615,77 | 513,04 |
| 844784 | 34,728287 | 32,529673 | 8074,25 | 1959,99 | 622,34 |
| 932143 | 34,728369 | 32,511013 | 3594,71 | 3198,38 | 1015,55 |
| 1036477 | 34,715140 | 32,513353 | 5192,41 | 2648,04 | 840,81 |
| 1036776 | 34,726534 | 32,516923 | 1707,73 | 1536,95 | 488,01 |
| 1072413 | 34,719673 | 32,527759 | 1570,40 | 773,22 | 245,51 |
| 782783 | 34,729383 | 32,530621 | 2772,84 | 1439,88 | 457,19 |
| 899505 | 34,731790 | 32,489819 | 929002,26 | 6119,55 | 1943,08 |
| 1153140 | 34,723070 | 32,516987 | 9235,33 | 1428,61 | 453,61 |
| 1250672 | 34,707857 | 32,541872 | 7245,80 | 2446,65 | 776,86 |

**Table A4**. Records of Cyprus Agricultural Payments Organisation (CAPO) for the year 2016, provided in the form of shape files — Cluster 4

| **Plot ID** | **Longitude**  **[˚]** | **Latitude**  **[˚]** | **Plot Area**  **[m²]** | **Greenhouse Area**  **[m²]** | **LDPE mass**  **[kg]** |
| --- | --- | --- | --- | --- | --- |
| 1012245 | 34,673560 | 32,687477 | 3040,76 | 1751,01 | 555,98 |
| 1157019 | 34,668198 | 32,729893 | 4359,92 | 1967,75 | 624,80 |

**Table A5**. Records of Cyprus Agricultural Payments Organisation (CAPO) for the year 2016, provided in the form of shape files — Cluster 5

| **Plot ID** | **Longitude**  **[˚]** | **Latitude**  **[˚]** | **Plot Area**  **[m²]** | **GreenhouseArea**  **[m²]** | **LDPE mass**  **[kg]** |
| --- | --- | --- | --- | --- | --- |
| 829989 | 34,833492 | 32,637320 | 35191,46 | 6663,58 | 2115,82 |
| 1309445 | 34,889906 | 32,625271 | 15723,20 | 7880,06 | 2502,08 |
| 867784 | 34,890991 | 32,623452 | 3384,86 | 1676,41 | 532,29 |
| 1014255 | 34,876240 | 32,808618 | 3773,91 | 478,83 | 152,04 |
| 890819 | 34,877325 | 32,805628 | 8026,84 | 1141,64 | 362,49 |
| 1069514 | 34,981114 | 32,463968 | 2411,84 | 1022,18 | 324,56 |
| 1293545 | 34,927238 | 32,540377 | 953,69 | 307,06 | 97,50 |
| 864059 | 34,980923 | 32,463592 | 4053,11 | 1351,82 | 429,23 |
| 1163452 | 34,927142 | 32,538034 | 265,09 | 165,32 | 52,49 |

**Table A6**. Records of Cyprus Agricultural Payments Organisation (CAPO) for the year 2016, provided in the form of shape files — Cluster 6

| **Plot ID** | **Longitude**  **[˚]** | **Latitude**  **[˚]** | **Plot Area**  **[m²]** | **GreenhouseArea**  **[m²]** | **LDPE mass**  **[kg]** |
| --- | --- | --- | --- | --- | --- |
| 866521 | 34,678720 | 32,953852 | 2016,51 | 1767,28 | 561,15 |
| 1148448 | 34,667741 | 32,925194 | 1542,04 | 1387,83 | 440,67 |
| 807753 | 34,675059 | 32,949295 | 4152,15 | 725,01 | 230,21 |
| 807854 | 34,659307 | 32,919288 | 19260,22 | 2124,51 | 674,57 |
| 1206642 | 34,654394 | 33,003117 | 24390,69 | 2680,33 | 851,06 |
| 808497 | 34,673906 | 32,913055 | 3341,46 | 1754,49 | 557,08 |
| 808572 | 34,664891 | 32,979607 | 3236,50 | 2912,85 | 924,89 |
| 881425 | 34,667213 | 32,950533 | 2197,83 | 1746,69 | 554,61 |
| 950527 | 34,659140 | 32,987162 | 23814,78 | 6851,37 | 2175,45 |
| 1039687 | 34,687191 | 32,910982 | 20302,35 | 5492,62 | 1744,02 |
| 1051578 | 34,664413 | 32,978889 | 7716,41 | 1377,98 | 437,54 |
| 1121489 | 34,664728 | 32,948485 | 3385,40 | 1562,23 | 496,04 |
| 867370 | 34,681249 | 32,926585 | 4063,65 | 1913,31 | 607,52 |
| 924931 | 34,671095 | 32,948031 | 1469,91 | 938,20 | 297,90 |
| 939115 | 34,660023 | 33,016621 | 6450,08 | 4785,17 | 1519,39 |
| 1148403 | 34,644099 | 32,918724 | 10576,40 | 1800,15 | 571,58 |
| 1162882 | 34,671607 | 33,021320 | 2620,07 | 1222,64 | 388,21 |
| 807822 | 34,681215 | 32,913260 | 3837,82 | 2810,34 | 892,34 |
| 808001 | 34,667245 | 32,951905 | 4304,87 | 2311,96 | 734,09 |
| 810137 | 34,679150 | 32,905053 | 5641,46 | 4891,56 | 1553,17 |
| 810306 | 34,674409 | 32,912815 | 3126,05 | 1477,18 | 469,03 |
| 1004527 | 34,665237 | 32,985874 | 20463,74 | 5603,06 | 1779,08 |
| 1011152 | 34,703484 | 32,986697 | 6554,29 | 3674,85 | 1166,84 |
| 1072663 | 34,659031 | 33,017075 | 12744,29 | 5118,44 | 1625,21 |
| 1099956 | 34,660391 | 32,905026 | 5173,14 | 4519,30 | 1434,97 |
| 1139443 | 34,685397 | 32,989970 | 5193,03 | 1663,03 | 528,04 |
| 806184 | 34,664666 | 32,945751 | 2915,26 | 658,69 | 209,15 |
| 807392 | 34,669135 | 32,951006 | 5926,91 | 2569,22 | 815,78 |
| 809659 | 34,671524 | 32,946949 | 3367,23 | 1541,21 | 489,36 |
| 946193 | 34,686405 | 32,989243 | 1836,92 | 936,04 | 297,21 |
| 1036563 | 34,661545 | 32,906971 | 26671,20 | 1867,15 | 592,86 |
| 1075554 | 34,667719 | 32,924664 | 2636,59 | 2319,35 | 736,44 |
| 773689 | 34,633795 | 32,959872 | 2236,79 | 2013,12 | 639,20 |
| 807191 | 34,659404 | 32,984270 | 5899,22 | 5286,82 | 1678,67 |
| 810143 | 34,671407 | 32,978898 | 5163,62 | 2312,81 | 734,36 |
| 923600 | 34,668739 | 32,977702 | 3645,85 | 2123,78 | 674,34 |
| 1062028 | 34,674532 | 32,903946 | 7622,04 | 4162,91 | 1321,81 |
| 810146 | 34,664797 | 32,945149 | 2500,94 | 1185,30 | 376,36 |
| 1058087 | 34,632577 | 32,935629 | 2492,88 | 324,32 | 102,98 |
| 1319977 | 34,660004 | 32,983995 | 6940,59 | 5717,12 | 1815,30 |
| 1320500 | 34,632858 | 32,935369 | 1485,61 | 956,68 | 303,77 |

**Table A7**. Records of Cyprus Agricultural Payments Organisation (CAPO) for the year 2016, provided in the form of shape files — Cluster 7

| **Plot ID** | | **Longitude**  **[˚]** | | **Latitude**  **[˚]** | **Plot Area**  **[m²]** | **GreenhouseArea**  **[m²]** | | **LDPE mass**  **[kg]** |
| --- | --- | --- | --- | --- | --- | --- | --- | --- |
| 818364 | | 34,745976 | | 33,154709 | 4039,27 | 1523,89 | | 483,87 |
| 933461 | | 34,738829 | | 33,191992 | 577,18 | 348,71 | | 110,72 |
| 948203 | | 34,767696 | | 33,191422 | 6014,46 | 4876,81 | | 1548,48 |
| 958597 | | 34,747198 | | 33,178977 | 2795,72 | 933,55 | | 296,42 |
| 1030719 | | 34,753312 | | 33,129910 | 1553,43 | 1178,50 | | 374,20 |
| 1031389 | | 34,736090 | | 33,190428 | 5501,90 | 1473,03 | | 467,72 |
| 1047390 | | 34,767123 | | 33,158384 | 2009,16 | 1496,34 | | 475,12 |
| 1201208 | | 34,755770 | | 33,140861 | 3112,48 | 2801,23 | | 889,45 |
| 805762 | | 34,760543 | | 33,139923 | 1958,64 | 1468,13 | | 466,16 |
| 809508 | | 34,764305 | | 33,156090 | 1713,09 | 1152,97 | | 366,09 |
| 911875 | 34,760963 | | 33,195968 | | 8639,96 | 1319,00 | 418,81 | |
| 949044 | 34,751575 | | 33,164791 | | 7048,23 | 1644,86 | 522,28 | |
| 966002 | 34,766078 | | 33,198315 | | 19476,54 | 15194,98 | 4824,71 | |
| 1001860 | 34,764200 | | 33,195921 | | 25141,26 | 16189,24 | 5140,41 | |
| 1030674 | 34,752896 | | 33,145413 | | 3513,13 | 1111,27 | 352,85 | |
| 1042563 | 34,774128 | | 33,154749 | | 4808,24 | 498,99 | 158,44 | |
| 1044587 | 34,763600 | | 33,161223 | | 4950,49 | 3172,53 | 1007,34 | |
| 1128765 | 34,760903 | | 33,188410 | | 15167,48 | 1095,74 | 347,92 | |
| 1129032 | 34,751027 | | 33,165984 | | 1397,54 | 1257,78 | 399,37 | |
| 832712 | 34,739723 | | 33,160486 | | 3220,83 | 2301,65 | 730,82 | |
| 880616 | 34,753089 | | 33,164968 | | 11081,08 | 3724,95 | 1182,75 | |
| 881545 | 34,773865 | | 33,166244 | | 4438,36 | 878,60 | 278,97 | |
| 931656 | 34,760103 | | 33,141627 | | 3354,22 | 1733,93 | 550,56 | |
| 954835 | 34,771124 | | 33,185963 | | 2081,41 | 1416,93 | 449,90 | |
| 994937 | 34,767705 | | 33,216252 | | 20630,95 | 5302,69 | 1683,71 | |
| 1113940 | 34,768975 | | 33,189064 | | 5137,10 | 4761,26 | 1511,80 | |
| 1147827 | 34,766175 | | 33,153913 | | 14756,65 | 3504,55 | 1112,76 | |
| 1235619 | 34,766283 | | 33,159286 | | 3086,13 | 2421,20 | 768,78 | |
| 831957 | 34,740137 | | 33,149674 | | 1640,89 | 1476,80 | 468,91 | |
| 855684 | 34,731370 | | 33,148487 | | 4143,95 | 3729,56 | 1184,21 | |
| 883843 | 34,768448 | | 33,149456 | | 8189,86 | 2904,89 | 922,36 | |
| 898073 | 34,747995 | | 33,174563 | | 2540,52 | 1985,31 | 630,38 | |
| 907337 | 34,751649 | | 33,151745 | | 6426,13 | 5432,40 | 1724,90 | |
| 1077944 | 34,757350 | | 33,178139 | | 11941,49 | 924,03 | 293,40 | |
| 1082429 | 34,780684 | | 33,248129 | | 6231,76 | 1542,68 | 489,83 | |
| 829448 | 34,767555 | | 33,164741 | | 13562,25 | 2165,75 | 687,67 | |
| 830765 | 34,763539 | | 33,160353 | | 8966,66 | 7943,72 | 2522,29 | |
| 881127 | 34,752522 | | 33,130138 | | 7800,11 | 6723,24 | 2134,76 | |
| 909566 | 34,760769 | | 33,140357 | | 6400,12 | 1685,45 | 535,16 | |
| 943094 | 34,753785 | | 33,129865 | | 2160,16 | 1258,95 | 399,74 | |
| 999382 | 34,764606 | | 33,157469 | | 5285,23 | 3187,18 | 1011,99 | |

**Table A7**. Records of Cyprus Agricultural Payments Organisation (CAPO) for the year 2016, provided in the form of shape files — Cluster 7 (cont’d)

| **Plot ID** | **Longitude**  **[˚]** | **Latitude**  **[˚]** | **Plot Area**  **[m²]** | **GreenhouseArea**  **[m²]** | **LDPE mass**  **[kg]** |
| --- | --- | --- | --- | --- | --- |
| 1009937 | 34,704805 | 33,088598 | 1560,75 | 485,65 | 154,21 |
| 1029278 | 34,739222 | 33,150477 | 1428,72 | 749,14 | 237,87 |
| 1058056 | 34,739562 | 33,150670 | 555,43 | 499,89 | 158,72 |
| 1071584 | 34,754355 | 33,149276 | 4690,66 | 2737,17 | 869,10 |
| 805829 | 34,767791 | 33,150965 | 6483,02 | 10056,31 | 3193,08 |
| 827563 | 34,743557 | 33,152930 | 1861,98 | 1449,19 | 460,15 |
| 829662 | 34,762110 | 33,196374 | 6229,57 | 5167,00 | 1640,63 |
| 837025 | 34,748710 | 33,168409 | 1219,66 | 716,31 | 227,44 |
| 875685 | 34,763224 | 33,145326 | 4480,89 | 3467,04 | 1100,86 |
| 969656 | 34,739837 | 33,159852 | 482,72 | 414,44 | 131,59 |
| 994195 | 34,750221 | 33,186361 | 5523,30 | 608,86 | 193,33 |
| 1031709 | 34,769729 | 33,188975 | 6964,21 | 5488,83 | 1742,81 |
| 1044350 | 34,772846 | 33,170823 | 10413,61 | 969,21 | 307,75 |
| 1153889 | 34,765685 | 33,180183 | 13745,42 | 21743,13 | 6903,88 |
| 805254 | 34,753961 | 33,130055 | 7813,18 | 3738,50 | 1187,05 |
| 819193 | 34,739569 | 33,150178 | 2963,43 | 726,63 | 230,72 |
| 849186 | 34,766548 | 33,160353 | 21765,76 | 6304,64 | 2001,85 |
| 920655 | 34,704558 | 33,088984 | 2154,83 | 914,51 | 290,37 |
| 934763 | 34,769409 | 33,188657 | 3355,71 | 3248,72 | 1031,53 |
| 982845 | 34,767127 | 33,187024 | 17598,12 | 1662,94 | 528,02 |
| 1043968 | 34,748981 | 33,190681 | 2865,00 | 859,59 | 272,94 |
| 805034 | 34,755933 | 33,210548 | 9694,55 | 3044,81 | 966,79 |
| 827936 | 34,764734 | 33,145815 | 9311,92 | 7874,28 | 2500,24 |
| 866558 | 34,753616 | 33,130687 | 5009,63 | 4298,25 | 1364,78 |
| 1033262 | 34,745447 | 33,151894 | 1463,95 | 1317,56 | 418,35 |
| 1066869 | 34,762658 | 33,156189 | 5307,67 | 1371,26 | 435,40 |
| 1106320 | 34,739016 | 33,192002 | 1088,85 | 464,48 | 147,48 |
| 1106518 | 34,755749 | 33,134391 | 3163,62 | 2674,23 | 849,12 |
| 1115200 | 34,765104 | 33,187122 | 16035,42 | 944,25 | 299,82 |

**Table A8**. Records of Cyprus Agricultural Payments Organisation (CAPO) for the year 2016, provided in the form of shape files — Cluster 8

| **Plot ID** | **Longitude**  **[˚]** | **Latitude**  **[˚]** | **Plot Area**  **[m²]** | **GreenhouseArea**  **[m²]** | **LDPE mass**  **[kg]** |
| --- | --- | --- | --- | --- | --- |
| 916522 | 34,858765 | 33,173368 | 1478,32 | 526,26 | 167,10 |
| 947914 | 34,843288 | 33,173164 | 1031,83 | 896,89 | 284,78 |
| 970428 | 34,858545 | 33,173490 | 1447,27 | 483,90 | 153,65 |
| 1001141 | 34,868345 | 33,075581 | 2949,70 | 471,06 | 149,57 |
| 1008065 | 34,867599 | 33,174718 | 1795,38 | 802,88 | 254,93 |
| 1124678 | 34,863831 | 33,086210 | 15720,90 | 6816,43 | 2164,35 |

**Table A8**. Records of Cyprus Agricultural Payments Organisation (CAPO) for the year 2016, provided in the form of shape files — Cluster 8 (cont’d)

| **Plot ID** | **Longitude**  **[˚]** | **Latitude**  **[˚]** | **Plot Area**  **[m²]** | **GreenhouseArea**  **[m²]** | **LDPE mass**  **[kg]** |
| --- | --- | --- | --- | --- | --- |
| 1140049 | 34,862285 | 33,094227 | 19014,65 | 3466,40 | 1100,65 |
| 1208292 | 34,859475 | 33,054262 | 7606,62 | 2302,33 | 731,04 |
| 1208302 | 34,858907 | 33,053073 | 5920,41 | 917,28 | 291,26 |
| 776379 | 34,868705 | 33,171196 | 2931,72 | 2209,85 | 701,67 |
| 808836 | 34,858190 | 33,173788 | 1813,53 | 508,18 | 161,36 |
| 844987 | 34,882667 | 33,184987 | 1738,18 | 323,59 | 102,75 |
| 908789 | 34,818090 | 33,144239 | 2647,58 | 1310,44 | 416,09 |
| 943697 | 34,858097 | 33,053962 | 6382,32 | 943,16 | 299,47 |
| 1040196 | 34,869600 | 33,075672 | 1634,81 | 457,45 | 145,25 |
| 1106909 | 34,866298 | 33,175401 | 7254,23 | 6190,29 | 1965,54 |
| 1127432 | 34,829900 | 33,061384 | 3453,98 | 802,51 | 254,81 |
| 1154491 | 34,868253 | 33,177008 | 3421,68 | 637,77 | 202,51 |
| 832771 | 34,861282 | 33,096030 | 7217,65 | 2220,65 | 705,10 |
| 936551 | 34,882297 | 33,185381 | 948,78 | 435,99 | 138,44 |
| 1008295 | 34,866999 | 33,174279 | 7464,62 | 13608,59 | 4321,00 |
| 1070031 | 34,830494 | 33,060847 | 3847,16 | 1097,67 | 348,53 |
| 821072 | 34,811071 | 33,175686 | 3565,40 | 795,61 | 252,62 |
| 831581 | 34,842016 | 33,120370 | 3273,11 | 1330,75 | 422,54 |
| 832184 | 34,876593 | 33,149618 | 2166,70 | 1725,00 | 547,72 |
| 961309 | 34,863965 | 33,082594 | 1171,60 | 395,17 | 125,47 |
| 1042514 | 34,808558 | 33,173110 | 4416,15 | 2311,28 | 733,88 |
| 1127575 | 34,817585 | 33,151597 | 5026,59 | 1869,14 | 593,49 |
| 785142 | 34,878791 | 33,086697 | 10995,25 | 3590,22 | 1139,97 |
| 848675 | 34,830615 | 33,167853 | 5207,73 | 1555,18 | 493,80 |
| 889043 | 34,867223 | 33,175026 | 1795,94 | 773,08 | 245,47 |
| 918419 | 34,851702 | 33,168628 | 1744,88 | 488,21 | 155,02 |
| 957599 | 34,811823 | 33,174710 | 3007,65 | 1540,29 | 489,07 |
| 1103753 | 34,831402 | 33,164752 | 2856,92 | 2539,05 | 806,20 |
| 830282 | 34,828540 | 33,024931 | 10566,31 | 3982,37 | 1264,48 |
| 930088 | 34,863704 | 33,082810 | 2596,01 | 680,17 | 215,97 |
| 997987 | 34,870528 | 33,075814 | 802,62 | 722,36 | 229,36 |
| 1034315 | 34,867539 | 33,173494 | 2085,04 | 822,70 | 261,23 |
| 819197 | 34,843751 | 33,050910 | 9178,01 | 4071,58 | 1292,81 |
| 828788 | 34,870509 | 33,168573 | 4182,42 | 2635,87 | 836,94 |
| 881936 | 34,834271 | 33,041459 | 3770,54 | 1085,69 | 344,73 |
| 916536 | 34,810529 | 33,174646 | 7185,08 | 700,79 | 222,51 |
| 930346 | 34,849588 | 33,096247 | 9243,67 | 4335,84 | 1376,72 |
| 1035855 | 34,841540 | 33,114713 | 3328,76 | 1063,09 | 337,55 |
| 1064683 | 34,865255 | 33,176745 | 2131,76 | 542,29 | 172,19 |
| 1094401 | 34,845438 | 33,172979 | 8499,86 | 7143,45 | 2268,19 |

**Table A8**. Records of Cyprus Agricultural Payments Organisation (CAPO) for the year 2016, provided in the form of shape files — Cluster 8 (cont’d)

| **Plot ID** | **Longitude**  **[˚]** | **Latitude**  **[˚]** | **Plot Area**  **[m²]** | **GreenhouseArea**  **[m²]** | **LDPE mass**  **[kg]** |
| --- | --- | --- | --- | --- | --- |
| 1183682 | 34,810334 | 33,164369 | 14542,81 | 3163,18 | 1004,37 |
| 1279284 | 34,863728 | 33,083364 | 2507,25 | 518,38 | 164,60 |
| 805617 | 34,811063 | 33,174873 | 4819,56 | 3116,86 | 989,67 |
| 815120 | 34,864413 | 33,081506 | 3417,32 | 1008,06 | 320,08 |
| 849307 | 34,826936 | 33,156678 | 1529,93 | 1252,33 | 397,64 |
| 877733 | 34,818666 | 33,147560 | 5866,67 | 3904,51 | 1239,76 |
| 899973 | 34,821226 | 33,157739 | 4080,06 | 676,60 | 214,84 |
| 964553 | 34,841168 | 33,116413 | 2006,46 | 505,71 | 160,57 |
| 1068846 | 34,817378 | 33,153109 | 3345,44 | 1214,00 | 385,47 |
| 1076662 | 34,816315 | 33,144778 | 3450,71 | 4038,91 | 1282,44 |
| 1151289 | 34,852301 | 33,169027 | 2880,73 | 607,97 | 193,04 |
| 1206874 | 34,836779 | 33,173944 | 3196,94 | 4632,02 | 1470,76 |
| 1222893 | 34,881303 | 33,185658 | 2969,36 | 1075,61 | 341,53 |

**Table A9**. Records of Cyprus Agricultural Payments Organisation (CAPO) for the year 2016, provided in the form of shape files — Cluster 9

| **Plot ID** | **Longitude**  **[˚]** | **Latitude**  **[˚]** | **Plot Area**  **[m²]** | **GreenhouseArea**  **[m²]** | **LDPE mass**  **[kg]** |
| --- | --- | --- | --- | --- | --- |
| 800505 | 34,761304 | 33,398451 | 1603,80 | 1443,42 | 458,31 |
| 809198 | 34,753468 | 33,403213 | 575,75 | 518,18 | 164,53 |
| 840811 | 34,760792 | 33,408032 | 3036,65 | 2108,56 | 669,51 |
| 920471 | 34,758362 | 33,311695 | 3224,43 | 1500,35 | 476,39 |
| 923541 | 34,747828 | 33,392430 | 9637,95 | 7477,16 | 2374,15 |
| 924006 | 34,737313 | 33,359662 | 4300,81 | 3524,66 | 1119,15 |
| 929825 | 34,737310 | 33,303612 | 883,27 | 719,10 | 228,33 |
| 932423 | 34,994634 | 33,924315 | 2534,09 | 1209,47 | 384,03 |
| 939513 | 34,733865 | 33,370224 | 4914,65 | 4423,19 | 1404,45 |
| 945220 | 34,795273 | 33,320921 | 11179,81 | 10061,83 | 3194,83 |
| 970537 | 34,738234 | 33,301350 | 2159,64 | 1805,71 | 573,35 |
| 1007151 | 34,736584 | 33,371147 | 3178,62 | 1814,68 | 576,20 |
| 1009644 | 34,749642 | 33,359143 | 1160,12 | 1044,11 | 331,53 |
| 1015177 | 34,752445 | 33,407650 | 5415,85 | 4874,27 | 1547,68 |
| 1016917 | 34,761508 | 33,419111 | 17728,08 | 4442,07 | 1410,44 |
| 1028324 | 34,762391 | 33,415499 | 5424,20 | 4817,94 | 1529,79 |
| 1028991 | 34,760645 | 33,369874 | 7401,57 | 2768,27 | 878,98 |
| 1093465 | 34,751965 | 33,313208 | 3570,34 | 2256,56 | 716,50 |
| 1099896 | 34,747073 | 33,317713 | 8257,53 | 3993,63 | 1268,06 |
| 1104771 | 34,738952 | 33,333280 | 3601,67 | 3280,63 | 1041,66 |
| 1110386 | 34,742139 | 33,371451 | 9945,39 | 3551,47 | 1127,66 |

**Table A9**. Records of Cyprus Agricultural Payments Organisation (CAPO) for the year 2016, provided in the form of shape files — Cluster 9 (cont’d)

| **Plot ID** | **Longitude**  **[˚]** | **Latitude**  **[˚]** | **Plot Area**  **[m²]** | **GreenhouseArea**  **[m²]** | **LDPE mass**  **[kg]** |
| --- | --- | --- | --- | --- | --- |
| 1118762 | 34,733927 | 33,357273 | 8164,38 | 7144,56 | 2268,54 |
| 1119028 | 34,735286 | 33,360343 | 11684,70 | 10221,78 | 3245,62 |
| 1133905 | 34,771225 | 33,338593 | 8905,42 | 4754,55 | 1509,66 |
| 1153258 | 34,734862 | 33,358588 | 10630,19 | 3850,36 | 1222,57 |
| 1207887 | 34,753494 | 33,317722 | 9033,38 | 2817,36 | 894,57 |
| 821458 | 34,734729 | 33,351260 | 7606,23 | 2580,52 | 819,37 |
| 850621 | 34,738538 | 33,357050 | 3293,81 | 2278,24 | 723,39 |
| 888239 | 34,753175 | 33,403191 | 2059,52 | 1853,57 | 588,55 |
| 898069 | 34,738589 | 33,315224 | 10722,48 | 9650,23 | 3064,14 |
| 918717 | 34,752157 | 33,409721 | 2465,68 | 1486,20 | 471,90 |
| 923535 | 34,741460 | 33,353916 | 12413,37 | 9920,76 | 3150,04 |
| 927661 | 34,738304 | 33,339036 | 13460,45 | 11345,43 | 3602,40 |
| 944578 | 34,753501 | 33,402181 | 845,64 | 761,07 | 241,66 |
| 978918 | 34,760898 | 33,417801 | 2135,00 | 1721,07 | 546,47 |
| 990262 | 34,736917 | 33,303467 | 1582,03 | 1173,00 | 372,45 |
| 997723 | 34,741540 | 33,363817 | 2996,29 | 1708,76 | 542,57 |
| 1009235 | 34,767646 | 33,428984 | 3478,91 | 3134,15 | 995,15 |
| 1010417 | 34,729678 | 33,326229 | 2515,75 | 1797,54 | 570,76 |
| 1015511 | 34,768273 | 33,425858 | 8941,69 | 8047,52 | 2555,25 |
| 1019778 | 34,741889 | 33,373322 | 7227,43 | 6504,68 | 2065,37 |
| 1045745 | 34,739334 | 33,357877 | 1213,21 | 1091,89 | 346,70 |
| 1056487 | 34,753251 | 33,408406 | 5222,96 | 2078,82 | 660,07 |
| 1061258 | 34,741455 | 33,355554 | 7417,49 | 5956,45 | 1891,29 |
| 1068296 | 34,739470 | 33,358210 | 1565,35 | 1408,82 | 447,33 |
| 1070486 | 34,741217 | 33,377299 | 9794,95 | 4255,29 | 1351,14 |
| 1073191 | 34,763624 | 33,414547 | 3958,87 | 1720,52 | 546,30 |
| 1107680 | 34,766582 | 33,429091 | 8557,06 | 1771,12 | 562,36 |
| 1121762 | 34,762399 | 33,417772 | 16549,28 | 8406,55 | 2669,25 |
| 1134011 | 34,746691 | 33,366703 | 8858,76 | 1609,48 | 511,04 |
| 1134142 | 34,753708 | 33,403366 | 2362,21 | 1358,33 | 431,30 |
| 1134609 | 34,736481 | 33,368795 | 6396,43 | 5657,71 | 1796,44 |
| 1196810 | 34,737045 | 33,316258 | 5507,79 | 4957,01 | 1573,95 |
| 799743 | 34,742489 | 33,351858 | 9068,24 | 5091,23 | 1616,57 |
| 824101 | 34,745950 | 33,369329 | 6876,51 | 6188,86 | 1965,09 |
| 837433 | 34,744707 | 33,309728 | 6427,96 | 5785,16 | 1836,91 |
| 863543 | 34,748143 | 33,365583 | 2681,20 | 2413,08 | 766,20 |
| 868103 | 34,741852 | 33,359285 | 2783,12 | 2504,81 | 795,33 |
| 884712 | 34,747637 | 33,391634 | 11816,60 | 9075,01 | 2881,50 |
| 901953 | 34,746467 | 33,370270 | 6261,31 | 3977,18 | 1262,83 |

**Table A9**. Records of Cyprus Agricultural Payments Organisation (CAPO) for the year 2016, provided in the form of shape files — Cluster 9 (cont’d)

| **Plot ID** | **Longitude**  **[˚]** | **Latitude**  **[˚]** | **Plot Area**  **[m²]** | **GreenhouseArea**  **[m²]** | **LDPE mass**  **[kg]** |
| --- | --- | --- | --- | --- | --- |
| 927033 | 34,753441 | 33,401976 | 352,83 | 317,55 | 100,83 |
| 929867 | 34,795480 | 33,329099 | 7847,51 | 5101,79 | 1619,92 |
| 938943 | 34,749820 | 33,358991 | 1678,56 | 1801,88 | 572,13 |
| 949801 | 34,729899 | 33,331171 | 8720,10 | 5104,71 | 1620,85 |
| 951919 | 34,747129 | 33,373314 | 7738,71 | 5723,37 | 1817,28 |
| 981573 | 34,743476 | 33,369894 | 12133,45 | 2948,82 | 936,31 |
| 985139 | 34,741837 | 33,358424 | 3639,37 | 2850,54 | 905,10 |
| 989275 | 34,739207 | 33,353502 | 10297,63 | 2498,00 | 793,17 |
| 1009238 | 34,735872 | 33,370675 | 1568,90 | 1400,33 | 444,63 |
| 1009555 | 34,764078 | 33,409836 | 3464,47 | 3118,02 | 990,04 |
| 1012352 | 34,734846 | 33,355203 | 4761,89 | 3628,21 | 1152,03 |
| 1015174 | 34,746709 | 33,387822 | 5346,30 | 2202,63 | 699,38 |
| 1015729 | 34,739969 | 33,349879 | 4809,99 | 1992,95 | 632,80 |
| 1017762 | 34,741278 | 33,361150 | 3911,57 | 1219,99 | 387,37 |
| 1020106 | 34,739447 | 33,373043 | 6030,48 | 4906,74 | 1557,99 |
| 1073247 | 34,752105 | 33,408378 | 4120,90 | 3708,81 | 1177,62 |
| 1076544 | 34,762907 | 33,421498 | 6856,25 | 6170,62 | 1959,30 |
| 1085489 | 34,745236 | 33,374647 | 9315,19 | 5720,20 | 1816,28 |
| 1092100 | 34,734331 | 33,356118 | 3568,64 | 1684,85 | 534,97 |
| 1117445 | 34,738352 | 33,373430 | 7024,88 | 2135,89 | 678,19 |
| 1118310 | 34,733544 | 33,358047 | 7927,34 | 5149,78 | 1635,16 |
| 1126104 | 34,747910 | 33,384452 | 3567,28 | 2469,09 | 783,98 |
| 1127350 | 34,752118 | 33,407347 | 901,33 | 811,20 | 257,57 |
| 1136894 | 34,742630 | 33,372072 | 8682,55 | 5646,30 | 1792,81 |
| 1155751 | 34,740045 | 33,357160 | 7367,80 | 5324,99 | 1690,79 |
| 1157220 | 34,754053 | 33,309714 | 3604,50 | 3244,05 | 1030,05 |
| 783712 | 34,748843 | 33,362374 | 4730,41 | 3105,45 | 986,04 |
| 802353 | 34,794391 | 33,325349 | 18893,82 | 4852,59 | 1540,80 |
| 835696 | 34,730927 | 33,331183 | 7377,69 | 1790,41 | 568,49 |
| 844174 | 34,736285 | 33,358880 | 11189,29 | 5623,82 | 1785,67 |
| 852344 | 34,737947 | 33,358276 | 3687,37 | 3310,05 | 1051,01 |
| 904932 | 34,753105 | 33,382501 | 4973,73 | 4476,35 | 1421,33 |
| 913308 | 34,742926 | 33,379130 | 3705,85 | 1603,67 | 509,20 |
| 938044 | 34,730661 | 33,330316 | 5648,18 | 1860,88 | 590,87 |
| 984919 | 34,741857 | 33,328915 | 10531,77 | 4421,90 | 1404,04 |
| 990626 | 34,742323 | 33,360445 | 17366,55 | 10834,07 | 3440,03 |
| 1011364 | 34,730219 | 33,325980 | 3157,82 | 2292,06 | 727,77 |
| 1012198 | 34,733560 | 33,366629 | 4215,73 | 2648,41 | 840,92 |
| 1015731 | 34,741034 | 33,349322 | 4664,72 | 2163,53 | 686,97 |

**Table A9**. Records of Cyprus Agricultural Payments Organisation (CAPO) for the year 2016, provided in the form of shape files — Cluster 9 (cont’d)

| **Plot ID** | **Longitude**  **[˚]** | **Latitude**  **[˚]** | **Plot Area**  **[m²]** | **GreenhouseArea**  **[m²]** | **LDPE mass**  **[kg]** |
| --- | --- | --- | --- | --- | --- |
| 1018085 | 34,750833 | 33,381363 | 7912,41 | 5716,54 | 1815,12 |
| 1020162 | 34,740354 | 33,374077 | 5172,69 | 4655,42 | 1478,19 |
| 1047310 | 34,746712 | 33,370663 | 4975,53 | 1886,29 | 598,93 |
| 1052815 | 34,747622 | 33,365068 | 4922,21 | 1897,68 | 602,55 |
| 1062042 | 34,751002 | 33,391174 | 8113,67 | 7302,30 | 2318,63 |
| 1100160 | 34,742020 | 33,349308 | 11266,79 | 5743,93 | 1823,81 |
| 1109723 | 34,753845 | 33,377716 | 7675,63 | 4290,76 | 1362,40 |
| 1164625 | 34,751638 | 33,376553 | 9483,27 | 7743,32 | 2458,66 |
| 1164713 | 34,742940 | 33,342097 | 11896,62 | 10706,95 | 3399,67 |
| 1196815 | 34,736326 | 33,317026 | 8865,35 | 851,90 | 270,49 |
| 809505 | 34,756072 | 33,304658 | 1582,47 | 1002,05 | 318,17 |
| 826420 | 34,742829 | 33,328959 | 10397,78 | 3586,14 | 1138,67 |
| 856966 | 34,749658 | 33,381396 | 5272,62 | 3214,50 | 1020,67 |
| 868617 | 34,732642 | 33,366829 | 4532,56 | 3849,34 | 1222,24 |
| 869432 | 34,737482 | 33,363424 | 5079,69 | 4030,74 | 1279,84 |
| 889663 | 34,743270 | 33,329720 | 2394,57 | 2155,11 | 684,29 |
| 905290 | 34,740382 | 33,356291 | 8507,16 | 5488,20 | 1742,61 |
| 922232 | 34,746203 | 33,318782 | 2256,22 | 1551,32 | 492,57 |
| 938504 | 34,755235 | 33,395360 | 3364,05 | 3027,64 | 961,34 |
| 944779 | 34,741856 | 33,376831 | 14127,62 | 3398,26 | 1079,02 |
| 974172 | 34,733138 | 33,356594 | 9872,35 | 8091,90 | 2569,34 |
| 985136 | 34,755461 | 33,407149 | 2085,76 | 1877,18 | 596,04 |
| 997714 | 34,739645 | 33,351705 | 16716,02 | 13445,90 | 4269,34 |
| 1088705 | 35,131284 | 32,519205 | 1916,45 | 1496,64 | 475,21 |
| 1128139 | 34,752223 | 33,409245 | 1100,02 | 990,02 | 314,35 |
| 1154288 | 34,739452 | 33,346015 | 18568,37 | 14427,42 | 4580,99 |
| 1154314 | 34,738292 | 33,351467 | 20505,46 | 8303,29 | 2636,46 |
| 1165692 | 34,746356 | 33,376806 | 4884,08 | 3816,92 | 1211,95 |
| 782310 | 34,753791 | 33,310566 | 3123,23 | 879,92 | 279,39 |
| 804057 | 34,739780 | 33,332864 | 6652,40 | 1868,10 | 593,16 |
| 874677 | 34,738941 | 33,359352 | 10129,74 | 8266,46 | 2624,77 |
| 885591 | 34,749915 | 33,386225 | 3301,87 | 1675,93 | 532,14 |
| 901383 | 34,753560 | 33,407066 | 2955,81 | 2195,95 | 697,26 |
| 901410 | 34,737775 | 33,372697 | 4841,74 | 3910,11 | 1241,54 |
| 906218 | 34,737427 | 33,360268 | 3149,61 | 2783,39 | 883,78 |
| 910655 | 34,747259 | 33,369882 | 3658,36 | 2833,20 | 899,60 |
| 918807 | 34,739172 | 33,352980 | 2631,35 | 2368,21 | 751,95 |
| 926780 | 34,743557 | 33,378620 | 1508,50 | 958,13 | 304,23 |

**Table A9**. Records of Cyprus Agricultural Payments Organisation (CAPO) for the year 2016, provided in the form of shape files — Cluster 9 (cont’d)

| **Plot ID** | **Longitude**  **[˚]** | **Latitude**  **[˚]** | **Plot Area**  **[m²]** | **GreenhouseArea**  **[m²]** | **LDPE mass**  **[kg]** |
| --- | --- | --- | --- | --- | --- |
| 938425 | 34,754467 | 33,309921 | 2720,59 | 2448,53 | 777,46 |
| 947173 | 34,752414 | 33,408233 | 750,62 | 675,56 | 214,50 |
| 948434 | 34,734224 | 33,362566 | 4369,65 | 2393,10 | 759,86 |
| 978783 | 34,738227 | 33,359965 | 4369,74 | 2218,04 | 704,27 |
| 989508 | 34,734264 | 33,362871 | 4276,51 | 5841,41 | 1854,76 |
| 1008322 | 34,739261 | 33,370305 | 2989,14 | 1186,67 | 376,79 |
| 1012510 | 34,742303 | 33,373011 | 6870,08 | 4602,83 | 1461,49 |
| 1015022 | 34,753421 | 33,377103 | 2335,08 | 1800,50 | 571,70 |
| 1017906 | 34,749855 | 33,380758 | 5530,42 | 5039,28 | 1600,07 |
| 1020108 | 34,740359 | 33,377651 | 15657,86 | 12278,50 | 3898,67 |
| 1020161 | 34,739822 | 33,373557 | 3798,06 | 4548,72 | 1444,31 |
| 1031829 | 34,744851 | 33,320181 | 7996,94 | 4348,63 | 1380,78 |
| 1037973 | 34,748354 | 33,386044 | 1961,80 | 907,38 | 288,11 |
| 1053048 | 34,764171 | 33,422108 | 3131,82 | 2798,34 | 888,53 |
| 1146720 | 34,734781 | 33,355753 | 3149,44 | 2453,64 | 779,08 |
| 1155736 | 34,743230 | 33,361322 | 13486,84 | 4348,23 | 1380,65 |
| 1157569 | 34,741915 | 33,370573 | 19639,29 | 17675,36 | 5612,28 |
| 889905 | 34,734153 | 33,350993 | 6940,24 | 6246,21 | 1983,30 |
| 902315 | 34,735401 | 33,351273 | 8285,66 | 3340,32 | 1060,62 |
| 904907 | 34,738414 | 33,356145 | 3962,53 | 3475,12 | 1103,42 |
| 911796 | 34,739339 | 33,362373 | 4511,86 | 1530,19 | 485,86 |
| 914033 | 34,767988 | 33,427074 | 2782,35 | 2504,12 | 795,11 |
| 921263 | 34,766148 | 33,400513 | 2259,87 | 1983,34 | 629,75 |
| 921580 | 34,751779 | 33,318518 | 8820,20 | 7938,18 | 2520,53 |
| 927991 | 34,739991 | 33,354148 | 9954,46 | 2969,85 | 942,99 |
| 932636 | 34,770164 | 33,339942 | 22677,94 | 10265,99 | 3259,66 |
| 933232 | 34,745832 | 33,373072 | 10867,57 | 4108,82 | 1304,63 |
| 938292 | 34,742916 | 33,377048 | 7367,59 | 5600,66 | 1778,32 |
| 974716 | 34,761113 | 33,401273 | 7732,37 | 6685,75 | 2122,86 |
| 999034 | 34,756214 | 33,403628 | 1382,52 | 1244,27 | 395,08 |
| 1012405 | 34,737025 | 33,368241 | 2715,92 | 2444,32 | 776,12 |
| 1014801 | 34,755213 | 33,394969 | 2837,14 | 2553,42 | 810,76 |
| 1015352 | 34,754004 | 33,402758 | 5066,51 | 3305,54 | 1049,57 |
| 1017757 | 34,739292 | 33,370747 | 1090,73 | 981,66 | 311,70 |
| 1018544 | 34,749913 | 33,379133 | 12382,52 | 7818,84 | 2482,64 |
| 1020109 | 34,741049 | 33,374103 | 3796,03 | 2990,13 | 949,43 |
| 1020160 | 34,745879 | 33,374226 | 6265,66 | 5639,10 | 1790,53 |
| 1031137 | 34,753901 | 33,402218 | 1515,60 | 1364,04 | 433,11 |

**Table A9**. Records of Cyprus Agricultural Payments Organisation (CAPO) for the year 2016, provided in the form of shape files — Cluster 9 (cont’d)

| **Plot ID** | **Longitude**  **[˚]** | **Latitude**  **[˚]** | **Plot Area**  **[m²]** | **GreenhouseArea**  **[m²]** | **LDPE mass**  **[kg]** |
| --- | --- | --- | --- | --- | --- |
| 1057198 | 34,736788 | 33,338158 | 5051,98 | 1360,69 | 432,05 |
| 1079065 | 34,741035 | 33,359377 | 3494,30 | 2086,51 | 662,51 |
| 1084859 | 34,741172 | 33,352011 | 17840,33 | 6987,46 | 2218,66 |
| 1086204 | 34,750605 | 33,378383 | 10016,09 | 2894,66 | 919,11 |
| 1096244 | 34,735043 | 33,357230 | 6603,40 | 3874,96 | 1230,38 |
| 1107837 | 34,748263 | 33,366323 | 3707,50 | 2077,21 | 659,56 |
| 1115975 | 34,736774 | 33,335668 | 2302,14 | 2071,92 | 657,88 |
| 1134637 | 34,739229 | 33,357598 | 849,20 | 764,28 | 242,67 |
| 1196834 | 34,737121 | 33,316735 | 8160,98 | 3071,88 | 975,38 |
| 1216158 | 34,743674 | 33,375597 | 8220,10 | 3536,33 | 1122,86 |
| 865496 | 34,757704 | 33,311820 | 1951,27 | 944,34 | 299,85 |
| 869882 | 34,737195 | 33,303215 | 1977,90 | 1258,21 | 399,51 |
| 880182 | 34,736882 | 33,361311 | 9212,18 | 2229,11 | 707,79 |
| 905551 | 34,741442 | 33,359232 | 2506,39 | 2190,06 | 695,39 |
| 911542 | 34,750723 | 33,380561 | 4082,71 | 3674,44 | 1166,71 |
| 913843 | 34,746222 | 33,369801 | 5287,18 | 1603,05 | 509,00 |
| 921151 | 34,753295 | 33,310665 | 1345,23 | 1210,71 | 384,42 |
| 983906 | 34,740249 | 33,369856 | 11219,07 | 1387,00 | 440,40 |
| 986140 | 34,738491 | 33,313835 | 28953,07 | 3271,74 | 1038,84 |
| 1012042 | 34,739853 | 33,362028 | 3756,46 | 3127,64 | 993,09 |
| 1012349 | 34,732935 | 33,361751 | 14056,90 | 6812,90 | 2163,23 |
| 1012351 | 34,740828 | 33,357327 | 5158,34 | 4394,40 | 1395,31 |
| 1014935 | 34,751055 | 33,377055 | 7498,75 | 5819,78 | 1847,90 |
| 1019970 | 34,741637 | 33,373720 | 4877,06 | 4125,24 | 1309,85 |
| 1025145 | 34,745841 | 33,365223 | 9571,54 | 4743,18 | 1506,05 |
| 1030462 | 34,757989 | 33,311025 | 2681,44 | 1265,48 | 401,82 |
| 1088250 | 34,745921 | 33,317604 | 11335,29 | 1913,85 | 607,69 |
| 1122453 | 34,741287 | 33,373874 | 4430,00 | 3475,54 | 1103,55 |
| 1132099 | 34,735819 | 33,357627 | 7761,15 | 6082,06 | 1931,18 |

**Table A10**. Records of Cyprus Agricultural Payments Organisation (CAPO) for the year 2016, provided in the form of shape files — Cluster 10

| **Plot ID** | **Longitude**  **[˚]** | **Latitude**  **[˚]** | **Plot Area**  **[m²]** | **GreenhouseArea**  **[m²]** | **LDPE mass**  **[kg]** |
| --- | --- | --- | --- | --- | --- |
| 930329 | 34,801358 | 33,476384 | 4568,54 | 4111,69 | 1305,54 |
| 999803 | 34,800681 | 33,476786 | 4586,80 | 4128,12 | 1310,76 |
| 1000980 | 34,831654 | 33,477882 | 6544,54 | 5890,08 | 1870,22 |
| 1037894 | 34,797881 | 33,484757 | 13439,39 | 12095,45 | 3840,55 |
| 1082857 | 34,852117 | 33,460568 | 1908,62 | 1717,76 | 545,42 |

**Table A10**. Records of Cyprus Agricultural Payments Organisation (CAPO) for the year 2016, provided in the form of shape files — Cluster 10 (cont’d)

| **Plot ID** | **Longitude**  **[˚]** | **Latitude**  **[˚]** | **Plot Area**  **[m²]** | **GreenhouseArea**  **[m²]** | **LDPE mass**  **[kg]** |
| --- | --- | --- | --- | --- | --- |
| 1131809 | 34,798020 | 33,478080 | 8395,70 | 5116,58 | 1624,62 |
| 1190768 | 34,826664 | 33,469808 | 1186,01 | 5881,75 | 1867,57 |
| 854579 | 34,788537 | 33,473646 | 2927,58 | 1060,50 | 336,73 |
| 859317 | 34,831309 | 33,472076 | 9696,64 | 3632,37 | 1153,35 |
| 873370 | 34,783913 | 33,475621 | 2877,62 | 1246,41 | 395,76 |
| 940924 | 34,779164 | 33,460727 | 4378,61 | 3337,76 | 1059,81 |
| 946928 | 34,789439 | 33,474244 | 4388,63 | 3949,77 | 1254,13 |
| 949807 | 34,798450 | 33,507778 | 9487,25 | 877,97 | 278,77 |
| 1009235 | 34,767646 | 33,428984 | 3478,91 | 3131,02 | 994,16 |
| 1015017 | 34,789364 | 33,473259 | 8952,49 | 1168,02 | 370,87 |
| 1015511 | 34,768273 | 33,425858 | 8941,69 | 8047,52 | 2555,25 |
| 1021237 | 34,799863 | 33,476843 | 1360,06 | 1224,06 | 388,66 |
| 1021598 | 34,799116 | 33,476686 | 15196,44 | 11388,73 | 3616,15 |
| 1022212 | 34,799212 | 33,477455 | 1925,35 | 1732,81 | 550,20 |
| 1022479 | 34,838057 | 33,466468 | 8724,93 | 7852,43 | 2493,30 |
| 1038594 | 34,837045 | 33,481205 | 12728,18 | 11455,36 | 3637,31 |
| 1043698 | 34,783314 | 33,479761 | 13226,37 | 6185,88 | 1964,14 |
| 1097328 | 34,788866 | 33,480751 | 31203,88 | 7243,03 | 2299,81 |
| 1107680 | 34,766582 | 33,429091 | 8557,06 | 7701,35 | 2445,33 |
| 1149941 | 34,793980 | 33,472317 | 1119,49 | 1007,54 | 319,91 |
| 1158228 | 34,797601 | 33,506994 | 17531,07 | 4103,40 | 1302,91 |
| 843241 | 34,832495 | 33,478576 | 21596,40 | 8347,54 | 2650,51 |
| 966623 | 34,831967 | 33,481498 | 3193,54 | 2874,19 | 912,61 |
| 970068 | 34,790330 | 33,467491 | 3608,90 | 1961,96 | 622,96 |
| 987455 | 34,789922 | 33,473996 | 5202,56 | 4176,91 | 1326,25 |
| 994336 | 34,832787 | 33,472500 | 4878,72 | 2439,97 | 774,74 |
| 1014056 | 34,789291 | 33,475079 | 5195,03 | 3184,49 | 1011,14 |
| 1060782 | 34,792237 | 33,469979 | 2830,96 | 1825,23 | 579,55 |
| 1085699 | 34,788064 | 33,476543 | 4781,41 | 4303,27 | 1366,37 |
| 1144066 | 34,796112 | 33,472303 | 11152,33 | 2821,86 | 896,00 |
| 1190897 | 34,827067 | 33,469639 | 853,39 | 768,05 | 243,87 |
| 799650 | 34,792911 | 33,475147 | 13804,87 | 12424,38 | 3944,99 |
| 801090 | 34,790484 | 33,466994 | 3020,61 | 2718,55 | 863,19 |
| 840409 | 34,786707 | 33,479411 | 14849,44 | 9588,42 | 3044,52 |
| 847560 | 34,778122 | 33,458236 | 15408,56 | 8832,82 | 2804,60 |
| 885434 | 34,826345 | 33,470913 | 1608,00 | 895,10 | 284,21 |
| 904941 | 34,772049 | 33,451418 | 12535,27 | 7791,74 | 2474,03 |
| 995891 | 34,794130 | 33,474565 | 25882,79 | 23294,51 | 7396,47 |
| 996182 | 34,826331 | 33,469016 | 3531,48 | 3178,33 | 1009,18 |
| 1012320 | 34,778743 | 33,459250 | 14912,59 | 8714,21 | 2766,94 |

**Table A10**. Records of Cyprus Agricultural Payments Organisation (CAPO) for the year 2016, provided in the form of shape files — Cluster 10 (cont’d)

| **Plot ID** | **Longitude**  **[˚]** | **Latitude**  **[˚]** | **Plot Area**  **[m²]** | **GreenhouseArea**  **[m²]** | **LDPE mass**  **[kg]** |
| --- | --- | --- | --- | --- | --- |
| 1020026 | 34,819532 | 33,475737 | 4665,41 | 1967,33 | 624,67 |
| 1021236 | 34,800909 | 33,476132 | 1193,35 | 1074,02 | 341,02 |
| 1048780 | 34,828236 | 33,455963 | 6398,74 | 4697,04 | 1491,40 |
| 1122271 | 34,851993 | 33,460225 | 2139,63 | 1925,67 | 611,44 |
| 1132021 | 34,789615 | 33,466807 | 1516,49 | 757,80 | 240,62 |
| 1158191 | 34,787494 | 33,460138 | 8261,02 | 5786,61 | 1837,37 |
| 821456 | 34,824132 | 33,493727 | 7151,06 | 6435,95 | 2043,54 |
| 822755 | 34,798010 | 33,478976 | 5726,19 | 3289,82 | 1044,59 |
| 844555 | 34,838024 | 33,464524 | 4960,75 | 4464,68 | 1417,62 |
| 901914 | 34,794989 | 33,479566 | 9256,02 | 4994,73 | 1585,93 |
| 954799 | 34,832675 | 33,480339 | 6941,63 | 1971,31 | 625,93 |
| 1015284 | 34,823849 | 33,484210 | 2659,75 | 2393,78 | 760,07 |
| 1016203 | 34,780311 | 33,463518 | 53501,14 | 6246,73 | 1983,46 |
| 1020443 | 34,802206 | 33,447146 | 40529,51 | 16554,41 | 5256,36 |
| 1090886 | 34,789853 | 33,467083 | 1640,87 | 542,22 | 172,17 |
| 1112250 | 34,793685 | 33,457556 | 22733,18 | 9862,90 | 3131,67 |
| 1221566 | 34,798914 | 33,475518 | 21508,51 | 11172,75 | 3547,57 |
| 805080 | 34,863803 | 33,464514 | 1487,99 | 1339,19 | 425,22 |
| 870864 | 34,820815 | 33,522298 | 25429,18 | 2662,45 | 845,38 |
| 907170 | 34,831446 | 33,481787 | 3644,62 | 2693,27 | 855,17 |
| 949129 | 34,784017 | 33,480099 | 12327,13 | 4664,46 | 1481,06 |
| 971079 | 34,798985 | 33,504996 | 19268,36 | 5617,12 | 1783,55 |
| 1014797 | 34,794359 | 33,460029 | 26556,47 | 7894,76 | 2506,75 |
| 1053313 | 34,769829 | 33,428463 | 7288,87 | 6559,99 | 2082,93 |
| 1056556 | 34,782006 | 33,478461 | 10820,94 | 5902,11 | 1874,04 |
| 1061085 | 34,786854 | 33,477529 | 10229,50 | 9206,55 | 2923,26 |
| 1081230 | 34,801173 | 33,517156 | 7274,88 | 2637,92 | 837,59 |
| 1088630 | 34,787732 | 33,477232 | 10811,36 | 9730,23 | 3089,54 |
| 1129708 | 34,825899 | 33,470436 | 2624,16 | 2361,74 | 749,90 |
| 1140299 | 34,819303 | 33,527466 | 4136,74 | 3723,07 | 1182,15 |
| 1182095 | 34,800736 | 33,472124 | 14551,69 | 2791,17 | 886,25 |
| 1210456 | 34,796994 | 33,484825 | 6370,07 | 5733,06 | 1820,36 |
| 810758 | 34,778886 | 33,460133 | 4139,07 | 2968,76 | 942,64 |
| 852190 | 34,786778 | 33,460771 | 5275,44 | 2931,83 | 930,91 |
| 854855 | 34,826271 | 33,470380 | 2114,77 | 1903,30 | 604,33 |
| 874739 | 34,799953 | 33,504668 | 5751,05 | 5175,94 | 1643,47 |
| 914033 | 34,767988 | 33,427074 | 2782,35 | 2504,12 | 795,11 |
| 976223 | 34,794211 | 33,480213 | 17174,19 | 12425,48 | 3945,34 |
| 1006608 | 34,800483 | 33,476409 | 1804,36 | 1623,92 | 515,63 |
| 1014501 | 34,774345 | 33,443628 | 23837,49 | 2474,67 | 785,76 |

**Table A10**. Records of Cyprus Agricultural Payments Organisation (CAPO) for the year 2016, provided in the form of shape files — Cluster 10 (cont’d)

| **Plot ID** | **Longitude**  **[˚]** | **Latitude**  **[˚]** | **Plot Area**  **[m²]** | **GreenhouseArea**  **[m²]** | **LDPE mass**  **[kg]** |
| --- | --- | --- | --- | --- | --- |
| 1014807 | 34,825954 | 33,470958 | 1626,94 | 908,87 | 288,58 |
| 1040709 | 34,788042 | 33,480764 | 2629,83 | 1759,95 | 558,82 |
| 1079334 | 34,778791 | 33,478149 | 7098,30 | 6388,47 | 2028,47 |
| 775057 | 34,833089 | 33,470128 | 2645,32 | 2380,78 | 755,95 |
| 870395 | 34,796425 | 33,473038 | 14125,89 | 976,39 | 310,02 |
| 870719 | 34,790076 | 33,476275 | 10355,98 | 7768,56 | 2466,67 |
| 931746 | 34,795038 | 33,478569 | 7023,36 | 6321,02 | 2007,05 |
| 1007387 | 34,791931 | 33,474625 | 5182,93 | 4664,63 | 1481,11 |
| 1011789 | 34,824461 | 33,493065 | 13899,13 | 9532,21 | 3026,67 |
| 1070511 | 34,794590 | 33,472333 | 8562,64 | 3421,25 | 1086,31 |
| 1072266 | 34,797123 | 33,465666 | 13312,84 | 6701,16 | 2127,75 |
| 1082916 | 34,793576 | 33,510369 | 21422,65 | 19280,39 | 6121,91 |
| 1091309 | 34,798135 | 33,446332 | 6958,22 | 2893,08 | 918,61 |
| 1113219 | 34,779803 | 33,460476 | 5078,52 | 1923,49 | 610,75 |
| 1133290 | 34,789665 | 33,480989 | 8927,53 | 8034,77 | 2551,20 |
| 1133993 | 34,795758 | 33,506863 | 24176,27 | 8579,49 | 2724,16 |
| 1158388 | 34,798168 | 33,472663 | 13381,51 | 3700,74 | 1175,06 |

**Table A11**. Records of Cyprus Agricultural Payments Organisation (CAPO) for the year 2016, provided in the form of shape files — Cluster 11

| **Plot ID** | **Longitude**  **[˚]** | **Latitude**  **[˚]** | **Plot Area**  **[m²]** | **GreenhouseArea**  **[m²]** | **LDPE mass**  **[kg]** |
| --- | --- | --- | --- | --- | --- |
| 780251 | 34,822827 | 33,575696 | 10532,27 | 1549,77 | 492,08 |
| 1014878 | 34,831424 | 33,562870 | 10520,27 | 9468,24 | 3006,36 |
| 829418 | 34,825778 | 33,545043 | 4414,57 | 3973,12 | 1261,54 |
| 1070135 | 34,826513 | 33,571110 | 2028,30 | 1825,47 | 579,62 |
| 1157497 | 34,826937 | 33,572107 | 12733,84 | 2714,98 | 862,06 |
| 1171301 | 34,871078 | 33,577090 | 14211,06 | 3939,63 | 1250,91 |
| 808354 | 34,842459 | 33,586059 | 5429,16 | 4886,24 | 1551,48 |
| 936048 | 34,845082 | 33,578399 | 4732,68 | 4259,42 | 1352,45 |
| 1014764 | 34,826217 | 33,567434 | 2102,84 | 1892,56 | 600,92 |
| 801193 | 34,874434 | 33,596739 | 36068,18 | 1611,27 | 511,61 |
| 896113 | 34,834889 | 33,590628 | 17839,53 | 3766,29 | 1195,87 |
| 897752 | 34,830207 | 33,573506 | 17504,49 | 6710,05 | 2130,58 |
| 1011313 | 34,830418 | 33,574830 | 12890,32 | 3871,64 | 1229,32 |
| 1014995 | 34,826096 | 33,559172 | 2551,87 | 6902,34 | 2191,63 |
| 1063662 | 34,825850 | 33,545983 | 4101,85 | 3691,66 | 1172,18 |
| 1201004 | 34,841481 | 33,591146 | 4963,59 | 2507,95 | 796,32 |
| 1224656 | 34,838897 | 33,560066 | 1049,13 | 944,22 | 299,81 |
| 776474 | 34,856591 | 33,585120 | 9790,11 | 8047,81 | 2555,34 |

**Table A11**. Records of Cyprus Agricultural Payments Organisation (CAPO) for the year 2016, provided in the form of shape files — Cluster 11 (cont’d)

| **Plot ID** | **Longitude**  **[˚]** | **Latitude**  **[˚]** | **Plot Area**  **[m²]** | **GreenhouseArea**  **[m²]** | **LDPE mass**  **[kg]** |
| --- | --- | --- | --- | --- | --- |
| 807475 | 34,826151 | 33,571398 | 2853,56 | 2568,21 | 815,46 |
| 810005 | 34,872058 | 33,592067 | 18924,57 | 4113,10 | 1305,99 |
| 1014890 | 34,839550 | 33,569835 | 7013,25 | 6311,93 | 2004,16 |
| 1224655 | 34,839092 | 33,559821 | 1479,26 | 1331,33 | 422,72 |

**Table A12**. Records of Cyprus Agricultural Payments Organisation (CAPO) for the year 2016, provided in the form of shape files — Cluster 12

| **Plot ID** | **Longitude**  **[˚]** | **Latitude**  **[˚]** | **Plot Area**  **[m²]** | **GreenhouseArea**  **[m²]** | **LDPE mass**  **[kg]** |
| --- | --- | --- | --- | --- | --- |
| 931765 | 35,014221 | 33,705563 | 11005,73 | 1395,27 | 191,88 |
| 972509 | 35,014483 | 33,704851 | 8894,43 | 5300,35 | 728,90 |
| 1018965 | 35,014812 | 33,703603 | 19366,81 | 4448,72 | 611,79 |
| 1086390 | 35,015237 | 33,704642 | 3267,12 | 2089,24 | 287,31 |
| 781355 | 35,034624 | 33,627085 | 2068,37 | 455,23 | 62,60 |
| 859058 | 35,009780 | 33,578870 | 16519,91 | 2200,27 | 302,58 |
| 953922 | 35,013715 | 33,705974 | 4651,65 | 4186,48 | 575,73 |
| 1118102 | 35,026279 | 33,605219 | 14166,49 | 10080,94 | 1386,33 |
| 886410 | 35,014804 | 33,704296 | 4010,48 | 2878,20 | 395,81 |

**Table A13**. Records of Cyprus Agricultural Payments Organisation (CAPO) for the year 2016, provided in the form of shape files — Cluster 13

| **Plot ID** | **Longitude**  **[˚]** | **Latitude**  **[˚]** | **Plot Area**  **[m²]** | **GreenhouseArea**  **[m²]** | **LDPE mass**  **[kg]** |
| --- | --- | --- | --- | --- | --- |
| 773253 | 35,055830 | 33,934236 | 2052,08 | 1627,02 | 516,61 |
| 790174 | 35,019327 | 33,928908 | 3988,51 | 3518,03 | 1117,05 |
| 804522 | 34,999676 | 33,920550 | 2359,48 | 1314,07 | 417,24 |
| 805117 | 35,015472 | 34,026346 | 1592,76 | 842,56 | 267,53 |
| 846574 | 35,004919 | 33,906256 | 25691,60 | 2129,05 | 676,01 |
| 884621 | 34,990557 | 34,047251 | 2905,05 | 2322,78 | 737,53 |
| 889171 | 35,062770 | 33,933073 | 1294,66 | 756,19 | 240,11 |
| 932494 | 34,994634 | 33,924315 | 2042,60 | 1566,49 | 497,39 |
| 933802 | 35,066882 | 33,939413 | 4291,53 | 2062,46 | 654,87 |
| 1005378 | 35,000264 | 33,918445 | 4180,98 | 2978,10 | 945,61 |
| 1022554 | 34,993873 | 33,899083 | 5085,94 | 3777,21 | 1199,34 |
| 1027625 | 34,996351 | 33,910101 | 3521,40 | 2602,72 | 826,42 |
| 1073217 | 35,045354 | 34,009752 | 8648,29 | 7092,16 | 2251,90 |
| 1087486 | 35,046755 | 34,017214 | 1991,84 | 1792,66 | 569,21 |
| 1099581 | 35,038771 | 33,989616 | 1527,26 | 1337,60 | 424,71 |
| 1099738 | 35,058346 | 33,945371 | 1669,28 | 1249,43 | 396,72 |
| 1134733 | 35,021392 | 33,932580 | 4359,41 | 3493,29 | 1109,19 |

**Table A13**. Records of Cyprus Agricultural Payments Organisation (CAPO) for the year 2016, provided in the form of shape files — Cluster 13 (cont’d)

| **Plot ID** | **Longitude**  **[˚]** | | **Latitude**  **[˚]** | | **Plot Area**  **[m²]** | | | **GreenhouseArea**  **[m²]** | | **LDPE mass**  **[kg]** |
| --- | --- | --- | --- | --- | --- | --- | --- | --- | --- | --- |
| 1146354 | 34,996124 | | 33,909252 | | 11571,32 | | | 10190,98 | | 3235,84 |
| 1146418 | 35,019123 | | 33,927769 | | 4460,82 | | | 2469,11 | | 783,99 |
| 1148310 | 35,002387 | | 33,919562 | | 12548,73 | | | 9845,23 | | 3126,06 |
| 1148612 | 35,019851 | | 33,930011 | | 9179,00 | | | 7853,21 | | 2493,55 |
| 1157596 | 35,036982 | | 34,033640 | | 3074,24 | | | 2617,93 | | 831,25 |
| 1189316 | 35,068668 | | 33,941784 | | 2491,49 | | | 1602,78 | | 508,91 |
| 1208387 | 35,016812 | | 33,931395 | | 4643,95 | | | 4247,66 | | 1348,72 |
| 1294497 | 35,051108 | | 33,885728 | | 11065,34 | | | 2524,94 | | 801,72 |
| 773501 | 35,017034 | | 33,968614 | | 5197,25 | | | 14636,33 | | 4647,33 |
| 796717 | 35,037626 | | 34,020602 | | 2724,07 | | | 2173,49 | | 690,13 |
| 802943 | 35,003506 | | 33,911355 | | 7566,30 | | | 4371,09 | | 1387,91 |
| 803203 | 35,027364 | | 33,902952 | | 26093,46 | | | 10919,17 | | 3467,06 |
| 806301 | 35,051119 | | 34,007594 | | 1386,11 | | | 1247,50 | | 396,11 |
| 873116 | 35,051603 | | 34,005005 | | 1089,34 | | | 980,40 | | 311,30 |
| 932318 | 35,067965 | | 33,944086 | | 3162,86 | | | 2846,57 | | 903,84 |
| 936930 | 35,044778 | | 34,010870 | | 1411,62 | | | 451,84 | | 143,47 |
| 1009843 | 35,023075 | | 33,921110 | | 7235,31 | | | 3628,63 | | 1152,16 |
| 1012073 | 34,986618 | | 33,906311 | | 5867,82 | | | 4608,58 | | 1463,32 |
| 1013405 | 35,022163 | | 33,933540 | | 7464,47 | | | 6431,72 | | 2042,20 |
| 1048889 | 35,059604 | | 34,000826 | | 6466,80 | | | 3782,56 | | 1201,04 |
| 1048960 | 35,047112 | | 34,019103 | | 1204,33 | | | 982,45 | | 311,95 |
| 1095517 | 34,998810 | | 33,910277 | | 5864,01 | | | 5273,87 | | 1674,56 |
| 1133673 | | 35,046764 | | 34,006459 | | 7494,20 | 1835,41 | | 582,78 | |
| 1148342 | | 34,990012 | | 34,047210 | | 3179,77 | 2766,33 | | 878,36 | |
| 1180896 | | 35,048180 | | 34,018193 | | 2759,29 | 810,99 | | 257,51 | |
| 796928 | | 34,990665 | | 33,913816 | | 2799,67 | 2519,70 | | 800,06 | |
| 800224 | | 35,063515 | | 34,002078 | | 593,57 | 532,44 | | 169,06 | |
| 822980 | | 35,035895 | | 34,036645 | | 1402,17 | 1153,74 | | 366,34 | |
| 876195 | | 35,068195 | | 33,952582 | | 1792,97 | 1623,30 | | 515,43 | |
| 876817 | | 35,070981 | | 33,894704 | | 13049,78 | 4032,24 | | 1280,32 | |
| 932677 | | 35,041861 | | 34,019493 | | 10400,56 | 8374,55 | | 2659,09 | |
| 990635 | | 35,063398 | | 34,001985 | | 445,85 | 401,27 | | 127,41 | |
| 1004017 | | 34,998277 | | 33,915186 | | 9791,18 | 5064,36 | | 1608,04 | |
| 1004200 | | 34,996186 | | 33,924755 | | 16866,99 | 6151,91 | | 1953,36 | |
| 1017868 | | 35,057988 | | 34,003138 | | 3142,63 | 1643,12 | | 521,72 | |
| 1020243 | | 35,062919 | | 33,927054 | | 10364,74 | 2469,12 | | 784,00 | |
| 1037742 | | 35,022113 | | 33,925709 | | 12297,99 | 9141,38 | | 2902,57 | |
| 1039333 | | 34,994142 | | 33,898722 | | 11063,52 | 4507,02 | | 1431,07 | |
| 1049491 | | 35,021164 | | 34,046583 | | 6061,19 | 4486,19 | | 1424,46 | |
| 1146121 | | 35,000937 | | 34,045049 | | 4124,32 | 3292,09 | | 1045,30 | |
| 1148097 | | 34,992005 | | 33,927768 | | 7090,22 | 6423,49 | | 2039,59 | |

**Table A13**. Records of Cyprus Agricultural Payments Organisation (CAPO) for the year 2016, provided in the form of shape files — Cluster 13 (cont’d)

| **Plot ID** | **Longitude**  **[˚]** | **Latitude**  **[˚]** | **Plot Area**  **[m²]** | **GreenhouseArea**  **[m²]** | **LDPE mass**  **[kg]** |
| --- | --- | --- | --- | --- | --- |
| 789553 | 34,994772 | 33,922814 | 14056,80 | 5045,09 | 1601,92 |
| 791797 | 35,014345 | 34,026270 | 3855,76 | 1219,27 | 387,14 |
| 795445 | 35,044445 | 33,941944 | 9263,62 | 7060,48 | 2241,84 |
| 796686 | 35,017032 | 33,969081 | 3100,65 | 2146,57 | 681,58 |
| 798146 | 34,981090 | 34,044783 | 9843,94 | 1573,84 | 499,73 |
| 799177 | 35,038018 | 34,034120 | 5459,61 | 3817,42 | 1212,11 |
| 800025 | 35,050497 | 34,013536 | 1075,95 | 968,36 | 307,47 |
| 800225 | 34,986539 | 34,017920 | 3097,76 | 2681,25 | 851,35 |
| 801618 | 35,063266 | 34,002353 | 1269,03 | 1142,13 | 362,65 |
| 801841 | 35,036448 | 34,036349 | 5787,95 | 1147,81 | 364,45 |
| 804596 | 35,047735 | 34,019594 | 4001,06 | 1752,52 | 556,46 |
| 1003109 | 34,997061 | 33,901407 | 7186,84 | 4834,29 | 1534,98 |
| 1013911 | 35,062547 | 33,936291 | 6179,20 | 483,35 | 153,47 |
| 1025937 | 35,056607 | 33,936610 | 7989,39 | 6642,18 | 2109,02 |
| 1027475 | 35,019923 | 33,923384 | 10197,05 | 5728,83 | 1819,02 |
| 1099653 | 35,045057 | 34,011048 | 2944,39 | 2476,63 | 786,38 |
| 1111015 | 35,046958 | 33,934770 | 1828,23 | 486,03 | 154,32 |
| 1121873 | 35,054585 | 34,002610 | 3988,76 | 1734,51 | 550,74 |
| 1202863 | 35,046367 | 34,011981 | 6137,99 | 3981,15 | 1264,09 |
| 772826 | 35,039243 | 33,991387 | 1444,27 | 1299,84 | 412,72 |
| 790471 | 35,039584 | 33,991518 | 1457,15 | 1311,43 | 416,41 |
| 790536 | 35,001370 | 34,045796 | 2676,32 | 2319,74 | 736,57 |
| 791029 | 34,996782 | 33,925329 | 514,03 | 432,35 | 137,28 |
| 795922 | 35,063666 | 34,002707 | 1099,13 | 984,69 | 312,66 |
| 803480 | 35,040228 | 33,993948 | 1147,77 | 867,81 | 275,55 |
| 804981 | 35,046980 | 34,011786 | 4253,12 | 2815,04 | 893,83 |
| 834702 | 35,022175 | 34,046874 | 1429,10 | 1237,59 | 392,96 |
| 898761 | 35,034589 | 34,018358 | 2394,44 | 611,60 | 194,20 |
| 931640 | 35,013436 | 34,026895 | 3195,86 | 1984,66 | 630,17 |
| 939824 | 34,994698 | 33,905309 | 11775,93 | 9796,43 | 3110,56 |
| 943188 | 35,064924 | 33,938944 | 4646,74 | 2875,27 | 912,95 |
| 982353 | 35,068542 | 33,952562 | 1299,53 | 1081,57 | 343,42 |
| 995745 | 34,979790 | 33,900477 | 3890,77 | 3146,54 | 999,09 |
| 1021407 | 35,067332 | 33,938224 | 1858,08 | 1261,22 | 400,46 |
| 1030901 | 35,068197 | 33,953111 | 3816,45 | 3343,10 | 1061,50 |
| 1048864 | 34,990034 | 33,913227 | 7193,50 | 5492,81 | 1744,08 |
| 1049758 | 34,993075 | 33,906045 | 5136,80 | 4612,57 | 1464,58 |
| 1085592 | 35,016037 | 33,982988 | 5235,64 | 1301,38 | 413,21 |
| 1190520 | 35,069032 | 33,941738 | 2971,05 | 2540,98 | 806,81 |
| 1269658 | 34,983249 | 34,059647 | 6001,83 | 1753,24 | 556,69 |
| 1269829 | 35,013966 | 34,025036 | 1590,39 | 1370,88 | 435,28 |

**Table A13**. Records of Cyprus Agricultural Payments Organisation (CAPO) for the year 2016, provided in the form of shape files — Cluster 13 (cont’d)

| **Plot ID** | **Longitude**  **[˚]** | **Latitude**  **[˚]** | **Plot Area**  **[m²]** | **GreenhouseArea**  **[m²]** | **LDPE mass**  **[kg]** |
| --- | --- | --- | --- | --- | --- |
| 796477 | 35,013729 | 34,027439 | 2010,11 | 1342,72 | 426,34 |
| 801815 | 35,047093 | 34,016926 | 2440,17 | 2267,65 | 720,02 |
| 851381 | 35,063987 | 34,003014 | 1999,37 | 1461,69 | 464,11 |
| 960206 | 34,993707 | 33,925623 | 7208,41 | 6144,30 | 1950,94 |
| 961756 | 34,991475 | 34,047242 | 4595,84 | 1659,00 | 526,77 |
| 1016817 | 35,062657 | 34,001800 | 4604,83 | 3498,72 | 1110,91 |
| 1027365 | 35,061226 | 33,932621 | 4081,46 | 971,67 | 308,52 |
| 1047572 | 35,071233 | 33,895911 | 8645,87 | 1590,19 | 504,92 |
| 1063427 | 35,023040 | 33,916553 | 5789,76 | 3052,95 | 969,37 |
| 1085431 | 35,018561 | 33,929247 | 11680,42 | 1386,05 | 440,10 |
| 1099488 | 34,999386 | 33,915450 | 1351,04 | 731,03 | 232,12 |
| 1106582 | 35,023702 | 33,917117 | 2289,31 | 1152,92 | 366,08 |
| 1123565 | 35,036393 | 34,020274 | 10723,97 | 5441,24 | 1727,70 |
| 1134021 | 35,055104 | 34,003159 | 2402,03 | 1770,68 | 562,23 |
| 798015 | 35,002336 | 33,918424 | 6046,54 | 4055,79 | 1287,79 |
| 800195 | 35,047447 | 34,019837 | 2842,18 | 1416,84 | 449,88 |
| 803353 | 35,051656 | 33,933706 | 4250,02 | 858,60 | 272,62 |
| 803566 | 35,061527 | 33,928869 | 7266,72 | 2710,20 | 860,54 |
| 804678 | 35,006944 | 34,056116 | 1283,94 | 1791,75 | 568,92 |
| 804866 | 35,021872 | 33,921549 | 10294,45 | 10149,18 | 3222,57 |
| 961284 | 34,995207 | 33,925081 | 4344,72 | 2682,91 | 851,88 |
| 992843 | 35,049614 | 34,016802 | 1580,09 | 1422,08 | 451,54 |
| 994983 | 34,994290 | 33,925689 | 1148,63 | 1033,77 | 328,24 |
| 1015747 | 35,059433 | 34,000756 | 5830,48 | 2651,40 | 841,87 |
| 1016612 | 35,051921 | 34,007463 | 939,76 | 832,88 | 264,46 |
| 1017683 | 35,062320 | 33,925717 | 8180,95 | 4744,75 | 1506,55 |
| 1051070 | 35,046075 | 34,009470 | 6403,07 | 1092,55 | 346,91 |
| 1051468 | 34,989866 | 34,047793 | 4680,71 | 4241,15 | 1346,65 |
| 1082703 | 34,998292 | 33,898492 | 14802,13 | 5556,32 | 1764,24 |
| 1097145 | 35,046789 | 34,018747 | 2296,36 | 911,28 | 289,35 |
| 1123531 | 35,043515 | 34,017507 | 7011,98 | 3568,57 | 1133,09 |
| 1158566 | 35,051590 | 33,955044 | 16829,48 | 9322,13 | 2959,96 |
| 1269433 | 35,001908 | 34,044807 | 3768,28 | 3178,94 | 1009,38 |
| 796298 | 34,990566 | 33,899375 | 10705,42 | 9600,86 | 3048,47 |
| 800045 | 35,060832 | 34,000105 | 4872,17 | 1943,33 | 617,05 |
| 800226 | 35,045531 | 34,010965 | 3038,08 | 1443,06 | 458,20 |
| 801558 | 35,006676 | 33,921691 | 1800,86 | 916,98 | 291,16 |
| 809255 | 35,050140 | 34,013767 | 1444,93 | 1314,93 | 417,52 |
| 1027402 | 35,036956 | 34,024790 | 2661,74 | 1606,80 | 510,19 |
| 1073420 | 35,068245 | 33,941895 | 5067,71 | 3736,15 | 1186,30 |
| 1082487 | 35,016457 | 33,983811 | 4197,91 | 3014,21 | 957,07 |

**Table A13**. Records of Cyprus Agricultural Payments Organisation (CAPO) for the year 2016, provided in the form of shape files — Cluster 13 (cont’d)

| **Plot ID** | **Longitude**  **[˚]** | **Latitude**  **[˚]** | **Plot Area**  **[m²]** | **GreenhouseArea**  **[m²]** | **LDPE mass**  **[kg]** |
| --- | --- | --- | --- | --- | --- |
| 1087199 | 35,050287 | 34,016904 | 1887,12 | 1698,41 | 539,28 |
| 1270304 | 35,041772 | 34,018044 | 6195,23 | 2855,51 | 906,68 |

**Table A14**. Records of Cyprus Agricultural Payments Organisation (CAPO) for the year 2016, provided in the form of shape files — Cluster 14

| **Plot ID** | **Longitude**  **[˚]** | **Latitude**  **[˚]** | **Plot Area**  **[m²]** | **GreenhouseArea**  **[m²]** | **LDPE mass**  **[kg]** |
| --- | --- | --- | --- | --- | --- |
| 772882 | 34,979803 | 33,795613 | 4878,36 | 3756,01 | 1192,61 |
| 797170 | 34,960339 | 33,892521 | 4453,07 | 3917,38 | 1243,85 |
| 790116 | 34,969144 | 33,830826 | 2718,10 | 757,88 | 240,64 |
| 791513 | 34,961745 | 33,891657 | 1315,09 | 1183,58 | 375,81 |
| 1037716 | 34,973770 | 33,815366 | 11619,06 | 8903,42 | 2827,02 |
| 803368 | 35,029453 | 33,872645 | 3924,58 | 2074,90 | 658,82 |
| 1023485 | 34,987509 | 33,807839 | 24079,70 | 8670,66 | 2753,11 |
| 1025174 | 34,987022 | 33,818857 | 3873,91 | 1800,44 | 571,68 |
| 1108538 | 35,009242 | 33,802578 | 3934,14 | 3536,11 | 1122,79 |
| 800034 | 35,014998 | 33,784237 | 29744,67 | 19940,40 | 6331,48 |
| 1020314 | 35,042014 | 33,846287 | 8444,64 | 4276,54 | 1357,89 |
| 1111870 | 35,020847 | 33,781769 | 12980,54 | 1439,60 | 457,10 |
| 792080 | 34,989578 | 33,801467 | 41314,89 | 9268,82 | 2943,04 |
| 797365 | 34,962070 | 33,891332 | 1361,88 | 1225,69 | 389,18 |
| 804433 | 35,028641 | 33,873861 | 10635,85 | 8190,46 | 2600,63 |
| 791202 | 34,969917 | 33,834032 | 680,08 | 472,94 | 150,17 |
| 797472 | 34,985606 | 33,834271 | 3925,07 | 3375,43 | 1071,77 |
| 806884 | 34,989466 | 33,809903 | 6441,86 | 5276,29 | 1675,33 |
| 879427 | 34,990247 | 33,809243 | 9356,34 | 8237,65 | 2615,62 |
| 997564 | 35,009180 | 33,801835 | 12282,23 | 11022,16 | 3499,76 |
| 1034132 | 35,009232 | 33,803240 | 8014,57 | 6872,75 | 2182,24 |
| 1098083 | 35,003758 | 33,793719 | 9978,68 | 5389,93 | 1711,41 |
| 798261 | 35,001409 | 33,815130 | 20461,98 | 9670,61 | 3070,61 |
| 801866 | 34,977174 | 33,881797 | 7372,42 | 3710,23 | 1178,07 |
| 950424 | 35,043629 | 33,846129 | 2093,71 | 1095,75 | 347,92 |
| 1000876 | 34,956945 | 33,870945 | 14763,73 | 3663,72 | 1163,30 |
| 1003851 | 35,042655 | 33,845418 | 152,84 | 137,99 | 43,82 |
| 1121515 | 35,055048 | 33,807312 | 21793,46 | 1768,35 | 561,49 |

**Table A15**. Records of Cyprus Agricultural Payments Organisation (CAPO) for the year 2016, provided in the form of shape files — Cluster 15

| **Plot ID** | **Longitude**  **[˚]** | **Latitude**  **[˚]** | **Plot Area**  **[m²]** | **GreenhouseArea**  **[m²]** | **LDPE mass**  **[kg]** |
| --- | --- | --- | --- | --- | --- |
| 775189 | 35,096720 | 33,094622 | 2411,40 | 1379,85 | 438,13 |
| 816026 | 35,093359 | 33,090485 | 1340,37 | 1059,48 | 336,41 |
| 864097 | 35,096782 | 33,074950 | 5301,20 | 1492,77 | 473,98 |
| 885747 | 35,049548 | 32,897327 | 16511,33 | 3408,71 | 1082,33 |
| 904726 | 35,048232 | 32,898884 | 10429,26 | 3155,71 | 1002,00 |
| 924595 | 35,093134 | 32,921258 | 32989,32 | 2310,65 | 733,68 |
| 1042636 | 35,065967 | 32,980308 | 1737,64 | 894,06 | 283,88 |
| 994771 | 35,048278 | 32,897493 | 4119,28 | 972,54 | 308,80 |
| 870986 | 35,067052 | 32,979492 | 16624,40 | 999,70 | 317,43 |
| 1071226 | 35,109956 | 33,089387 | 1049,58 | 553,79 | 175,84 |
| 1113946 | 35,097530 | 32,968337 | 3070,81 | 1451,11 | 460,76 |
| 923108 | 35,064343 | 32,979054 | 11684,25 | 1167,69 | 370,76 |
| 831970 | 35,075824 | 32,934284 | 4820,95 | 931,12 | 295,65 |
| 839250 | 35,107436 | 33,089984 | 2544,44 | 865,44 | 274,79 |

**Table A16**. Records of Cyprus Agricultural Payments Organisation (CAPO) for the year 2016, provided in the form of shape files — Cluster 16

| **Plot ID** | **Longitude**  **[˚]** | **Latitude**  **[˚]** | **Plot Area**  **[m²]** | **GreenhouseArea**  **[m²]** | **LDPE mass**  **[kg]** |
| --- | --- | --- | --- | --- | --- |
| 967377 | 35,056908 | 33,271921 | 1128,90 | 876,03 | 278,16 |
| 845263 | 35,035194 | 33,364061 | 3892,15 | 3244,05 | 1030,05 |
| 940215 | 35,053451 | 33,438234 | 20921,92 | 3355,03 | 1065,29 |
| 940234 | 35,052999 | 33,437194 | 14234,44 | 10263,00 | 3258,71 |
| 1044059 | 35,058213 | 33,272585 | 1665,25 | 1498,73 | 475,88 |
| 1059473 | 35,057304 | 33,272669 | 6188,42 | 857,26 | 272,20 |
| 1103560 | 35,057283 | 33,273709 | 2183,25 | 1629,49 | 517,40 |
| 972213 | 35,035050 | 33,362988 | 1460,00 | 1288,61 | 409,16 |
| 976656 | 35,057758 | 33,271396 | 12706,45 | 1846,08 | 586,17 |
| 1147069 | 35,035037 | 33,364765 | 5354,14 | 4745,33 | 1506,74 |
| 877825 | 35,057172 | 33,271430 | 3187,81 | 934,68 | 296,78 |
| 894603 | 35,034801 | 33,362870 | 1170,74 | 1032,28 | 327,77 |
| 945402 | 35,059170 | 33,272300 | 2489,98 | 2240,98 | 711,56 |
| 1008237 | 35,052375 | 33,438054 | 12518,66 | 4155,06 | 1319,31 |
| 1008236 | 35,055431 | 33,438415 | 92071,83 | 7177,83 | 2279,10 |
| 1057710 | 35,056800 | 33,270441 | 10764,56 | 2317,96 | 736,00 |
| 934839 | 35,058815 | 33,270766 | 8152,23 | 3808,32 | 1209,22 |
| 976657 | 35,058085 | 33,270894 | 3037,32 | 1444,23 | 458,57 |
